# Environmental regulation of male fertility is mediated through Arabidopsis transcription factors bHLH89, 91, and 10

**DOI:** 10.1093/jxb/erad480

**Published:** 2023-12-08

**Authors:** Jordan K Robson, Alison C Tidy, Stephen G Thomas, Zoe A Wilson

**Affiliations:** Division of Plant & Crop Sciences, School of Biosciences, University of Nottingham, Sutton Bonington Campus, Loughborough, Leicester LE12 5RD, UK; Division of Plant & Crop Sciences, School of Biosciences, University of Nottingham, Sutton Bonington Campus, Loughborough, Leicester LE12 5RD, UK; Sustainable Soils and Crops, Rothamsted Research, Harpenden, Hertfordshire AL5 2JQ, UK; Division of Plant & Crop Sciences, School of Biosciences, University of Nottingham, Sutton Bonington Campus, Loughborough, Leicester LE12 5RD, UK; Ohio State University, USA

**Keywords:** Anther, Arabidopsis, bHLH, fertility, light, male sterility, pollen, tapetum

## Abstract

Formation of functional pollen and successful fertilization rely on the spatial and temporal regulation of anther and pollen development. This process responds to environmental cues to maintain optimal fertility despite climatic changes. Arabidopsis transcription factors basic helix–loop–helix (bHLH) 10, 89, and 91 were previously thought to be functionally redundant in their control of male reproductive development, however here we show that they play distinct roles in the integration of light signals to maintain pollen development under different environmental conditions. Combinations of the double and triple bHLH10,89,91 mutants were analysed under normal (200 μmol m^–2^ s^–1^) and low (50 μmol m^–2^ s^–1^) light conditions to determine the impact on fertility. Transcriptomic analysis of a new conditionally sterile *bhlh89,91* double mutant shows differential regulation of genes related to sexual reproduction, hormone signal transduction, and lipid storage and metabolism under low light. Here we have shown that bHLH89 and bHLH91 play a role in regulating fertility in response to light, suggesting that they function in mitigating environmental variation to ensure fertility is maintained under environmental stress.

## Teaser

Transcription factors bHLH89 and bHLH91 play a role in regulating fertility in Arabidopsis in response to light, suggesting that they function in mitigating environmental variation to ensure fertility is maintained under environmental stress.

## Introduction

Formation of functional pollen and subsequent successful fertilization rely upon the spatial and temporal regulation of anther and pollen development. The tapetum plays an important role in this through the regulation of meiotic progression and the coordination, synthesis, and secretion of the pollen wall. Genetic control of Arabidopsis tapetum development involves a series of complex interacting regulatory networks containing feedback and feedforward loops (Zhu *et al.*, 2011; Ferguson *et al.*, 2017) in which several transcription factors are known to play key roles including the pivotal basic helix–loop–helix (bHLH) transcription factors DYSFUNCTIONAL TAPETUM1 (DYT1; Zhang *et al*., 2006) and ABORTED MICROSPORES (AMS; Sorensen *et al.*, 2003). DYT1 regulates AMS via DEFECTIVE IN TAPETAL DEVELOPMENT AND FUNCTION 1 (TDF1), a putative R2R3 MYB transcription factor (Zhu *et al.*, 2008; Gu *et al*., 2014), and AMS in turn influences expression of the PHD-finger motif protein MALE STERILE1 (MS1; Wilson *et al.*, 2001) through MYB103/MS188, another R2R3 MYB protein (Zhang *et al.*, 2007; Lu *et al.*, 2020). DYT1 and AMS have also been shown to interact with three additional bHLH proteins: bHLH10, bHLH89, and bHLH91 (Xu *et al.*, 2010; Feng *et al.*, 2012).

Previous studies demonstrated that bHLH10, 89, and 91 are functionally redundant in anther development, regulating control of callose deposition and pollen wall formation (Zhu *et al.*, 2015). The *bhlh* single mutants have been shown to be fully fertile and morphologically indistinguishable from wild-type (WT) Col-0, while the double and triple *bhlh* mutants have increasingly defective anther development. The double mutants *bhlh089 bhlh091* and *bhlh089 bhlh010* have been reported as having reduced numbers of mature pollen grains, whereas the triple *bhlh089 bhlh010 amiR-bHLH091* mutant is completely male sterile, with smaller anthers and shorter filaments (Zhu *et al.*, 2015). Transverse sections of the triple *bhlh* mutant anthers and early anthers of the *bhlh089 bhlh010* double mutant showed abnormally large and disorganized tapetal cells with increased vacuolation at stage 6 (microspore meiosis) and notably thinner callose walls which exhibited delayed degradation. In *bhlh089 bhlh091* and late *bhlh089 bhlh010* anthers, the tapetal cell size was observed to be uneven and the tapetum layer less organized than those of the WT (Zhu *et al.*, 2015). These tapetum defects are similar to those observed in the male-sterile *dyt1* and *ams* mutants, which have large, vacuolated tapetum layers (Sorensen *et al.*, 2003; Zhang *et al.*, 2006; Xu *et al.*, 2010); *dyt1* mutant meiocytes have thinner callose walls as in the *bhlh089 bhlh010* mutant (Zhang *et al.*, 2006), and the *ams* mutant has irregular sized tapetum cells which exhibit delayed degradation (Xu *et al.*, 2010). Beyond tapetal defects, the *bhlh089 bhlh010* double mutant pollen has thinner and more uneven exine and intine layers (Lai *et al.*, 2022).

bHLH89, 91, and 10 were previously shown to interact with AMS and DYT1 in yeast two-hybrid (Y2H) and glutathione *S*-transferase (GST) pull-down assays (Xu *et al.*, 2010; Feng *et al.*, 2012). Models indicate that the dimerization of DYT1 and AMS with bHLH10, 89, and 91 may be competitive, with DYT1–bHLH complexes forming earlier (Ferguson *et al.*, 2017), and in DYT1’s case bHLH dimerization is required for its function and nuclear localization (Cui *et al.*, 2016).

Despite the high sequence similarity and functional redundancy indicated by the *bhlh89*, *91*, and *10* mutant phenotypes, these bHLHs have been shown to play distinct roles in activating downstream transcription (Cui *et al.*, 2016; Lai *et al.*, 2022). Different combinations of bHLH89/91/10 with DYT1 showed differential transcriptional activation; the DYT1–bHLH89 heterodimer, for example, bound to the MYB35 promoter to strongly activate expression, whereas DYT1–bHLH10 and DYT1–bHLH91 heterodimers showed lower activation capabilities (Cui *et al.*, 2016).

Recent studies have shown that *bhlh089 bhlh010* and *bhlh089 bhlh091* double mutants exhibit conditional sterility in response to high temperatures (Fu *et al.*, 2020) In this study we characterize new alleles of *bhlh89,91* and *bhlh89,10* double mutants and show that *bHLH89*, *bHLH10*, and *bHLH91* are not fully redundant but have distinct impacts on fertility. They present novel responses to environmental conditions with a distinct light-sensitive male sterility phenotype, manifesting in an extended sterility phenotype in early flowers under low light conditions.

Light is known to influence male reproductive development in terms of day length and the duration of light exposure, but additionally light intensity has been shown to affect the initiation of flowering via phytochrome-mediated pathways involving PHYTOCHROME INTERACTING FACTORS (PIFs), a class of bHLH proteins (Franklin, 2008; Wit *et al.*, 2016). There have been few studies into the effects of light on reproductive development beyond floral transition; to date, only MYB33, MYB65, YET ANOTHER KINASE1 (YAK1), and HEAT SHOCK PROTEIN-RELATED (HSPR) have a reported link between light and male fertility in Arabidopsis (Millar and Gubler, 2005; Huang *et al*., 2017; Yang *et al.*, 2020). *yak1* mutants have shorter siliques and reduced seed number, and this phenotype is intensified under low light intensity (50 µmol m^–2^ s^–1^; Huang *et al*., 2017). Likewise, the *hspr* mutant flowers later, with reduced pollen viability and seed set in low light conditions (20–60 µmol m^–2^ s^–1^; Yang *et al.*, 2020). Anthers from the Arabidopsis GAMYB-Like *myb33 myb65* double mutant exhibit a hypertrophic tapetum and a pre-meiotic block in microspore development, resulting in plants that are partially male sterile. However, full fertility is restored to *myb33 myb65* under high light intensities (330 µmol m^–2^ s^–1^) and low temperatures (16 °C; Millar and Gubler, 2005).

Here we show that *bhlh89,91* and *bhlh89,10* mutants exhibit increased sterility in response to not only high temperatures but also low light. When grown under low light conditions, both *bhlh* double mutants show prolonged initial sterility characterized by minor changes in tapetum phenology and striking defects in pollen wall patterning. We further report transcriptomic changes in pre-mitotic flower buds under changing light intensities which suggest an important role for tapetum bHLHs in maintaining pollen fertility under adverse conditions.

## Materials and methods

### Plant materials and growth conditions

*Arabidopsis thaliana* Columbia (Col-0) (WT) ecotype and mutants were sown onto a 3:1 mix of Levington M3 compost:vermiculite with T34 biological control. However, recent experiments looking at the effects of higher temperature and light levels were carried out on a 3:1 mix containing Klasmman BP substrate instead of Levington M3.

T-DNA insertion lines were obtained from SALK and GABI-KAT collections at the Nottingham Arabidopsis Stock Centre. Lines used in this study were GK-055H02 for *bhlh10*, SALK-123106 for *bhlh89*, and GK-345C06 for *bhlh91.* These lines were crossed to produce the double mutants *bhlh89,10* and *bhlh89,91.* The triple mutant line *bhlh089 bhlh010 amiR-bHlH091* was obtained from Zhu *et al.* (2015).

Plants were grown at 21 °C (16 h days under fluorescent lighting, with a 4:4:1 ratio of red:green:blue light). At the onset of flowering, just before bolting, plants were moved to different environmental treatments. In normal light conditions, plants were illuminated with a photosynthetic photon flux density (PPFD) of 205 (±8.6 SD) µmol m^–2^ s^–1^, while the low light plants received 53 (±5) µmol m^–2^ s^–1^ PPFD through use of net shading. For high temperature treatment, plants were transferred to 28 °C. Light availability and spectra were recorded by a Li-180 Spectrometer (Licor) at the plant base. Trays were rotated weekly to minimize localized environmental effects.

### Phenotypic characterization

Fully developed siliques were measured from the pedicel contact point to their tip. Lengths were recorded over developmental time from the earliest silique (position 1) to the most recent.

### Imaging anther cross-sections

Inflorescences were vacuum infiltrated for 30 min and fixed overnight at 4 °C in 4% (v/v) paraformaldehyde in phosphate-buffered saline (PBS) then washed twice in PBS. Dehydration was carried out by an ethanol series (2 × 10 min in 50, 70, 90, and 100% v/v) and 2 × 10 min in 100% propylene oxide. Inflorescences were soaked for 30 min in 3:1 propylene oxide:TAAB Low Viscosity (TLV) resin solution and overnight in 1:1 propylene oxide:TLV resin. Samples were transferred into 100% TLV resin for 2.5 h twice, and then embedded in fresh TLV resin to polymerize at 60 °C for 2 d.

Semi-thin (500 nm) sections were prepared using a Leica EM UC6 Ultramicrotome fitted with a glass knife, and stained with toluidine blue in 1% (v/v) sodium borate, before imaging by bright-field microscopy using a Leica DM5000b.

For TEM, inflorescences were fixed overnight at 4 °C in 3% (v/v) glutaraldehyde in 0.1 M cacodylate buffer then washed and stored in 0.1 M cacodylate buffer. Samples were stained for 1 h in 1% (v/v) osmium tetroxide, washed twice in dH_2_O, and soaked for 1 h in the dark at 4 °C in 2% (v/v) aqueous uranyl acetate. Dehydration and resin embedding was then carried out as above.

Ultra-thin sections (90 nm) were cut with a Leica EM UC6 Ultramicrotome fitted with a diamond knife, and then flattened using a heated element and collected onto 200-mesh carbon-coated copper grids (EM Resolutions Ltd) and left to dry. Sample matrices were placed on a droplet of saturated uranyl acetate in 50% (v/v) ethanol for 5 min then vigorously washed twice in 50% (v/v) ethanol and once in dH_2_O. Grids were subsequently placed on a droplet of Reynold’s lead citrate, surrounded by sodium hydroxide pellets, for at least 5 min and then washed twice in dH_2_O. Ultra-thin sections were then imaged using an FEI Tecnai 12 BioTwin transmission electron microscope.

### Scanning electron microscopy

Dehisced anthers were imaged using the FEI Quanta 650 ESEM at University of Nottingham’s Nanoscale and Microscale Research Centre (nmRC).

### Staging of buds

Buds were imaged using a Stemi SV6 stereomicroscope (Zeiss) and measured from their tip to the pedicel using ZEN lite software (Zeiss). Isolated anthers were stained in 3 µg ml^–1^ DAPI in 50% (v/v) glycerol, pressed to release microspores, and left for >16 h in darkness at 4 °C to allow the stain to infiltrate the microspores. Stages of meiosis were observed using the A4 fluorescence cube of a Leica DM5000b microscope.

### Quantitative reverse transcription–PCR

Total RNA was extracted from inflorescences using an RNeasy Mini Kit with on column DNase treatment (Qiagen). A 1 µg aliquot of cDNA was synthesized from the RNA using SuperScript III Reverse Transcriptase (ThermoFisher) and tested for gDNA contamination with the –RT controls by PCR using RedTaq DNA Polymerase (VWR) with reference PP2A3 primers (Supplementary Table S1).

Quantitative reverse transcription–PCR (qRT–PCR) was performed using PowerUp SYBR Green Master (Applied Biosystems) and the primers listed in Supplementary Table S1, with expression changes detected by a LightCycler 480 (Roche Life Science). To calculate relative gene expression (E), Ct values were adjusted according to pre-calculated primer efficiencies (x) and then normalized to the housekeeping gene PP2A3 (H) using the equation: E=100×^H–Ct^, since PP2A3 expression was stable across different experimental conditions (Supplementary Fig. S1). Expression data are from 3–6 biological replicates, with each biological replicate consisting of the mean of three technical replicates; statistical testing was carried out by two-way ANOVA with Tukey post-hoc tests.

### RNA isolation and RNA-sequencing

RNA was isolated from pre-mitotic buds, containing anther stages 1–10, from four factor groups (*bhlh89,91* Low, *bhlh89,91* Norm, Col-0 Low, and Col-0 Norm), each with four biological replicates, using the RNeasy Kit (Qiagen) and sequenced using the BGISEQ-500 platform (BGI).

Clean reads were mapped to *Arabidopsis thaliana* Reference Transcript Dataset 2 (AtRTD2; Zhang *et al.*, 2017) using the Salmon tool on UseGalaxy.eu (Patro *et al.*, 2017). The 3D-RNASeq app was used for differential expression analysis (Calixto *et al.*, 2018; Guo *et al.*, 2019). Read counts and transcripts per million reads (TPMs) were generated using tximport R package version 1.10.0 and the length scaled TPM method (Soneson *et al.*, 2016). Low expressed transcripts and genes were filtered based on analysis of the data mean variance trend, with an optimal filter of low expression set at ≥1 of the 16 samples with a count per million reads (CPM) ≥1. The TMM method was used to normalize gene and transcript read counts to log_2_-CPM (Bullard *et al.*, 2010). Batch effects were checked for using a principal component analysis (PCA) plot and showed that the RNA-seq dataset did not have distinct batch effects. The Limma R package was used for 3D expression comparison of contrast groups (Law *et al.*, 2014; Ritchie *et al.*, 2015); the log_2_ fold change (log_2_FC) of gene/transcript abundance was calculated based on contrast groups, and the significance of expression changes was determined using *t*-test. *P*-values of multiple testing were adjusted by the Benjamini–Hochberg method to correct the false discovery rate (FDR) (Benjamini and Yekutieli, 2001). A gene/transcript was significantly differentially expressed in a contrast group if it had an FDR-adjusted *P*-value (*q*) <0.01 and log_2_FC ≥1.

Hierarchical clustering was used to partition the differentially expressed genes (DEGs) into 10 clusters based on Euclidean distance and the ward.D clustering algorithm, and heatmaps were generated using ComplexHeatmap R package version 1.20.0 (Gu *et al*., 2016). Gene Ontology (GO) and Kyoto Encyclopaedia of Genes and Genomes (KEGG) pathway enrichment analysis was performed using the online Database for Annotation, Visualization and Integrated Discovery (DAVID) v6.8 (https://david.ncifcrf.gov/home.jsp) (Huang *et al*., 2009a, b). Mapping of expression changes to KEGG pathways was carried out with Pathview in R (Luo and Brouwer, 2013).

## Results

### Fertility of *bhlh* double mutants is reduced in low light

T-DNA insertion lines of *bhlh89* (SALK-123106), *bhlh91* (GK-345C06-016233), and *bhlh10* (GK-055H02-012479) were crossed to obtain double knockout mutants: *bhlh89,91* and *bhlh89,10*, whilst the triple mutant *bhlh089 bhlh010 amiR-bHLH091* was provided by Zhu *et al*. (2015) (Supplementary Fig. S2).

**Fig. 1.**
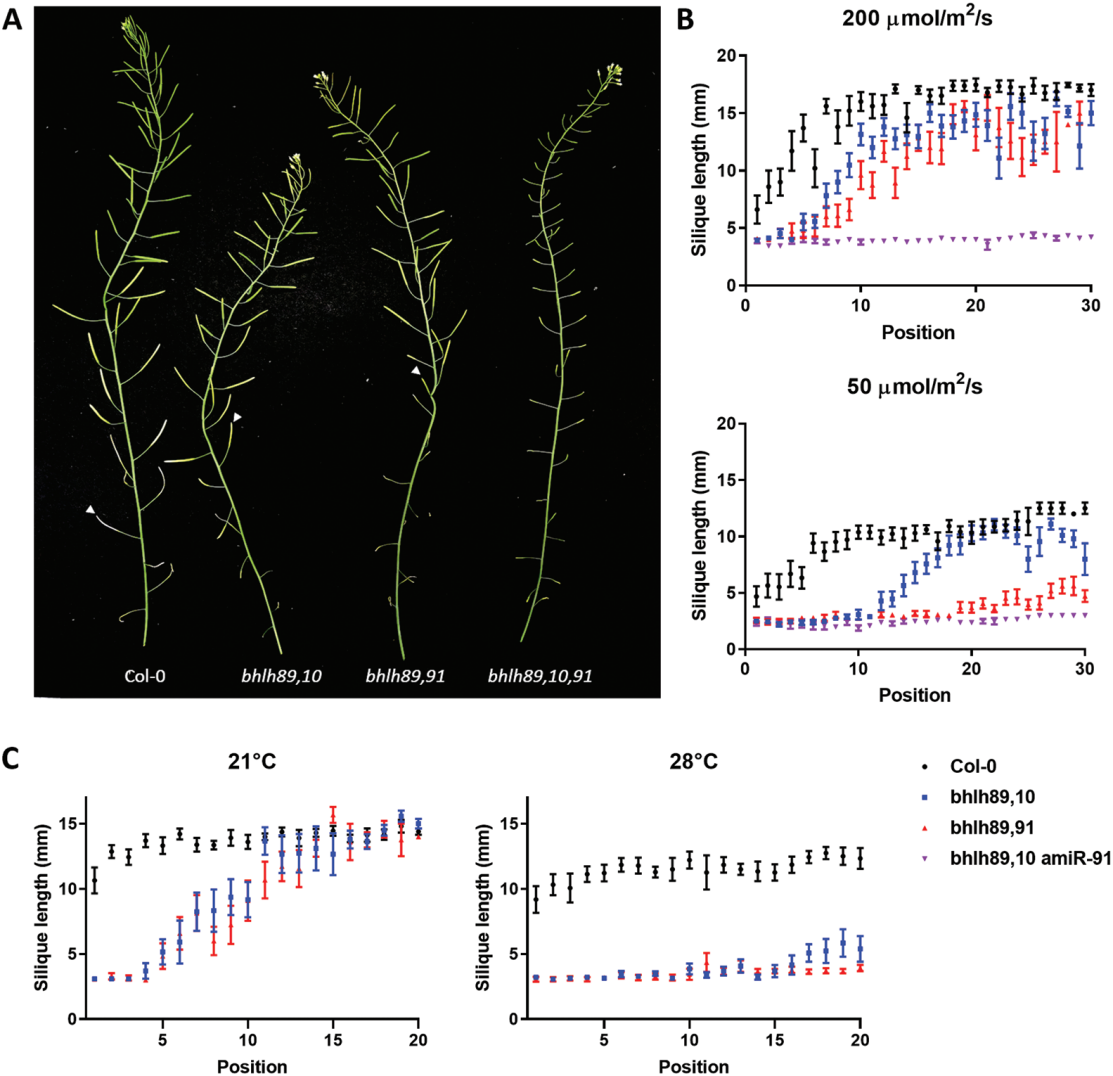
Characterization of *bhlh* double and triple mutants. (A) Delayed onset of fertility in *bhlh* double mutants compared with Col-0, grown in normal conditions (200 µmol m^–2^ s^–1^), is highlighted by a white arrow at the first fertile silique; the triple *bhlh89,10,91* mutant remained completely sterile throughout development. (B and C) The effect of low light (50 µmol m^–2^ s^–1^) (B) and 28 °C heat (C) on fertility, measured by silique length across the flowering stem; both elicit prolonged initial sterility in the *bhlh* double mutants. Mean values and the SE are plotted.

Fertility of the lines was determined through silique length measurements which correlated to seed set (Supplementary Fig. S3). As previously reported (Zhu *et al*. 2015), under normal growth conditions single mutants developed as WT (Supplementary Fig. S4), and the triple mutant was fully sterile with short empty siliques; however, both double mutants exhibited partial sterility in early flowers with later restoration of fertility indicated by long, filled siliques (Fig. 1A). Typically, the first 4–5 siliques to develop in Col-0 (WT) are shorter with reduced seed set, whilst normal seed set and silique development occurs in the subsequent flowers. However, both the *bhlh89,10* and *bhlh89,91* mutants exhibited a delay in this onset of fertility, with stunted silique development until approximately position 10 in the *bhlh89,10* mutant and position 18 in the *bhlh89,91* mutant (Fig. 1B). Notably, siliques of both *bhlh* double mutants remained significantly shorter due to reduced seed set than Col-0 when full fertility has been ‘recovered’ [ANOVA, *F*(2,279)=309, *P*<0.0001], both achieving, on average, a final length of 13–14 mm compared with 17 mm in Col-0.

To assess the effect of light on fertility, we grew Col-0, and *bhlh89,10*, *bhlh89,91*, and *bhlh089,bhlh010,amiR-bHLH091* mutants under standard conditions until bolting (light levels of 200 µmol m^–2^ s^–1^), at which point they were maintained under these conditions or transferred to low intensity light (50 µmol m^–2^ s^–1^), with an identical spectrum (Supplementary Fig. S5).

The fertility of all lines tested was reduced under low light conditions, except the *bhlh089,bhlh010*,*amiR-bHLH091* triple mutant, which was already fully sterile under normal light conditions and was unaffected by reducing the light intensity (Fig. 1B). In the WT, a final silique length of 12 mm on average was observed in low light, compared with 17 mm in normal light conditions (Fig. 1B), equating to a 28% reduction in maximum seed set (Supplementary Fig. S3). In low light, the *bhlh89,10* mutant remained sterile for longer than in normal light conditions, but fertility was eventually restored by approximately flower position 20, compared with position 10 under normal light, reaching an average final length of 10 mm. However, in low light, there was only minimal rescue of fertility observed in the *bhlh89,91* mutant, which was not restored to levels observed in Col-0 [ANOVA, *F*(3,444)=173, *P*<0.0001]. While the *bhlh10* single mutant showed no increase in initial sterility in response to low light, the *bhlh89* and *bhlh91* single mutants exhibited a minor delay in onset of fertility compared with Col-0; however, WT levels of fertility were established by position 7 (Supplementary Fig. S4). Under increased light levels (300 µmol m^–2^ s^–1^), both *bhlh89,10* and *bhlh89,91* double mutants were more fertile, with a reduced period of initial sterility (Supplementary Fig. S6).

Since a recent study by Fu *et al.* (2020) showed that a *bhlh089 bhlh010* mutant was hypersensitive to heat, we further tested the impact of heat stress on rescue of fertility in our *bhlh* double mutants and found that a 28 °C moderate heat stress elicited a similar, but more severe, prolonged period of sterility to that of low light, with some later recovery in the *bhlh89,10* mutant leading to seed production that was not seen in *bhlh89,91* (Fig. 1C, D).

### Microspore development in *bhlh* double mutants is unaffected by light until pollen mitosis I

Inflorescences were harvested from plants growing in normal and low light conditions; individual buds were measured and microspores isolated and stained with DAPI to assess how light and the bHLH mutations affect microspore development. Bud length has previously been shown to be indicative of developmental progression (Higgins *et al.*, 2014). No significant difference was found between the lengths of Col-0 and *bhlh* mutant buds at any stage of microspore development in either light condition (Supplementary Fig. S7A, B, restricted maximum likelihood *F*=0.1, *P*>0.05). However, in normal versus low light conditions, bicellular stage Col-0 buds were significantly smaller in low light conditions than those grown in normal light (Supplementary Fig. S7C; *P*=0.02, df=12, *t*=2.6). No significant difference was recorded in the length of buds from plants grown under different light conditions in the *bhlh89,91* or *bhlh89,10* double mutants; however, in low light conditions, microspore development aborted after the bicellular microspore stage in *bhlh89,91* buds and after the single microspore stage in early *bhlh89,10* buds (Supplementary Fig. S7D–F).

### *bhlh* double mutants exhibit defective microspore development

**Fig. 2.**
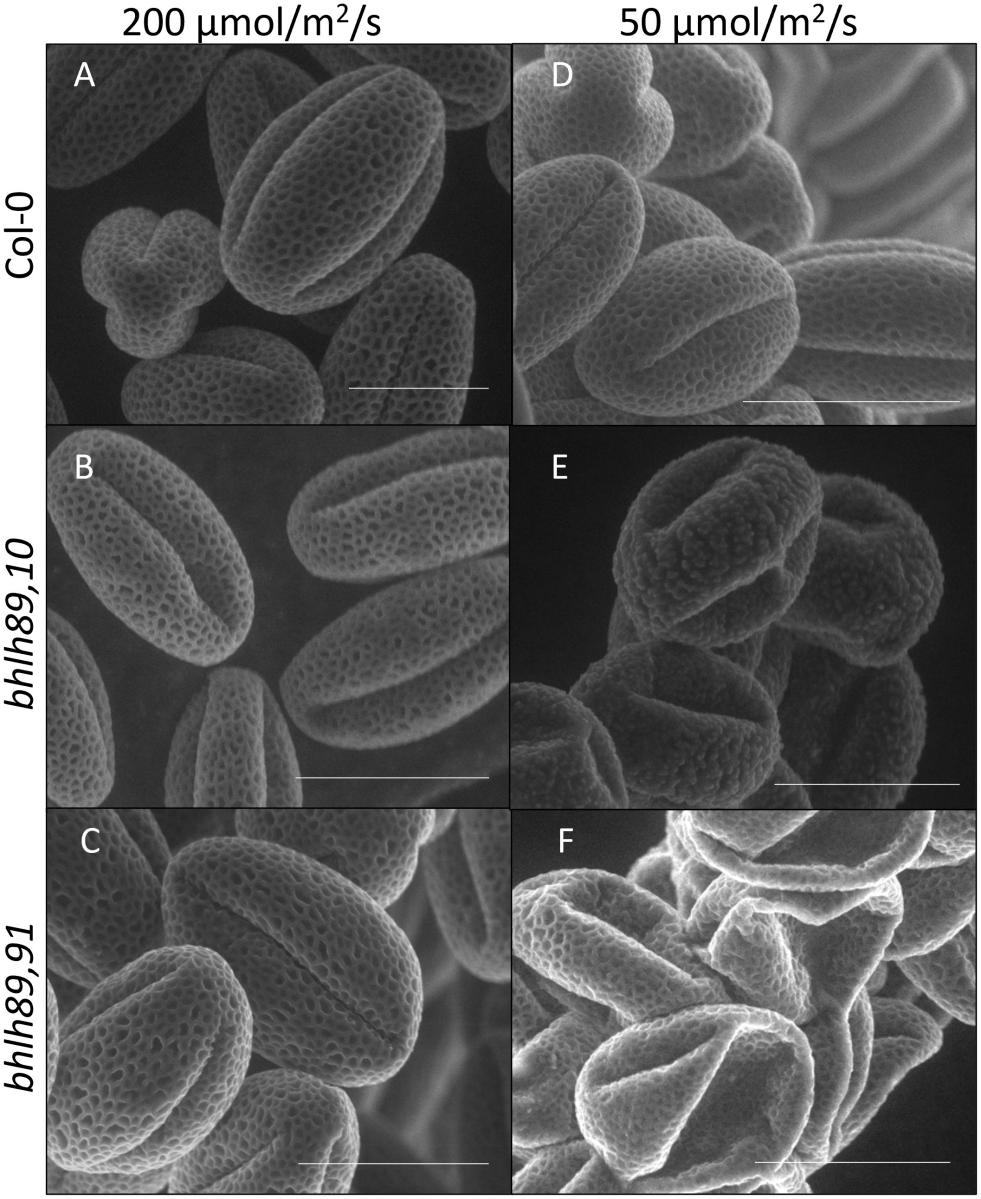
SEM images of pollen surfaces of Col-0 and *bhlh* double mutants grown under normal (200 µmol m^–2^ s^–1^; A–C) and low (50 µmol m^–2^ s^–1^; D–F) light conditions. Scale bar=20 µm.

SEM of mature pollen revealed that normal pollen shape and exine patterning were maintained in Col-0 in both light conditions (Fig. 2A, D). However, whilst *bhlh* double mutant pollen develops like Col-0 under normal light conditions (Fig. 2A–C), under low light both *bhlh89,10* and *bhlh89,91* double mutants show defective pollen exine patterning (Fig. 2E, F). The *bhlh89,10* mutant showed highly irregular exine deposits and abnormal indentation, whereas *bhlh89,91* exine deposition was regular but pollen was misshapen, collapsed, and formed aggregated clumps, and was non-viable according to Alexander staining (Supplementary Fig. S8). TEM imaging suggests that these pollen wall defects originate pre-mitosis, with unicellular *bhlh89,91* microspores showing a callose wall that had not fully degraded and reduced electron-dense baculae (Supplementary Fig. S9).

**Fig. 3.**
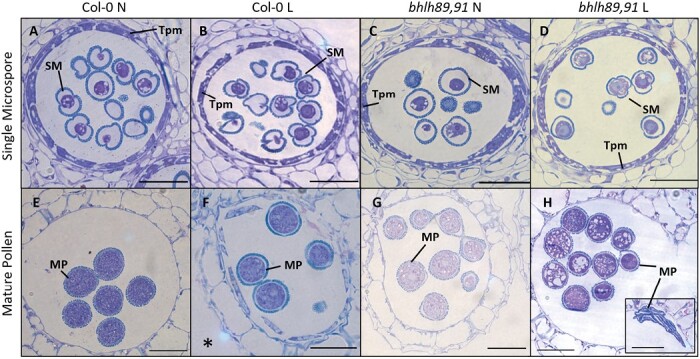
Semi-thin sections of Col-0 and *bhlh89 bhlh91* double mutant anthers, in normal (N) and low (L) light conditions in single microspores (A–D) and mature pollen (E–H). The *bhlh* mutant shows accumulation of globular lipid bodies in mature pollen especially under low light, and pollen collapse (H, inset). *Note that Col-0 L mature pollen (F) is viewed at a slightly earlier stage, prior to complete degradation of the tapetum, compared with (E), (G). and (H). Tpm, tapetum; SM, single microspore, MP, mature pollen. Scale bars=50 μm.

To further explore developmental differences in *bhlh89,91* versus Col-0 low light response, semi-thin anther sections were stained with toluidine blue (Fig. 3; Supplementary Fig. S10). At the tetrad stage, the tapetum of *bhlh89,91* appeared to be more vacuolated and separated from cell walls in comparison with Col-0 in low light (Supplementary Fig. S10). At the tricellular stage, just prior to anthesis, microspores which developed under low light in the *bhlh89,91* mutant were also more vacuolated, and a significant number of microspores were completely collapsed (Fig. 3H). Based upon Nile red staining, there were no discernible differences in lipid accumulation within tricellular pollen (Supplementary Fig. S11).

### bHLH transcription is reduced in low light

**Fig. 4.**
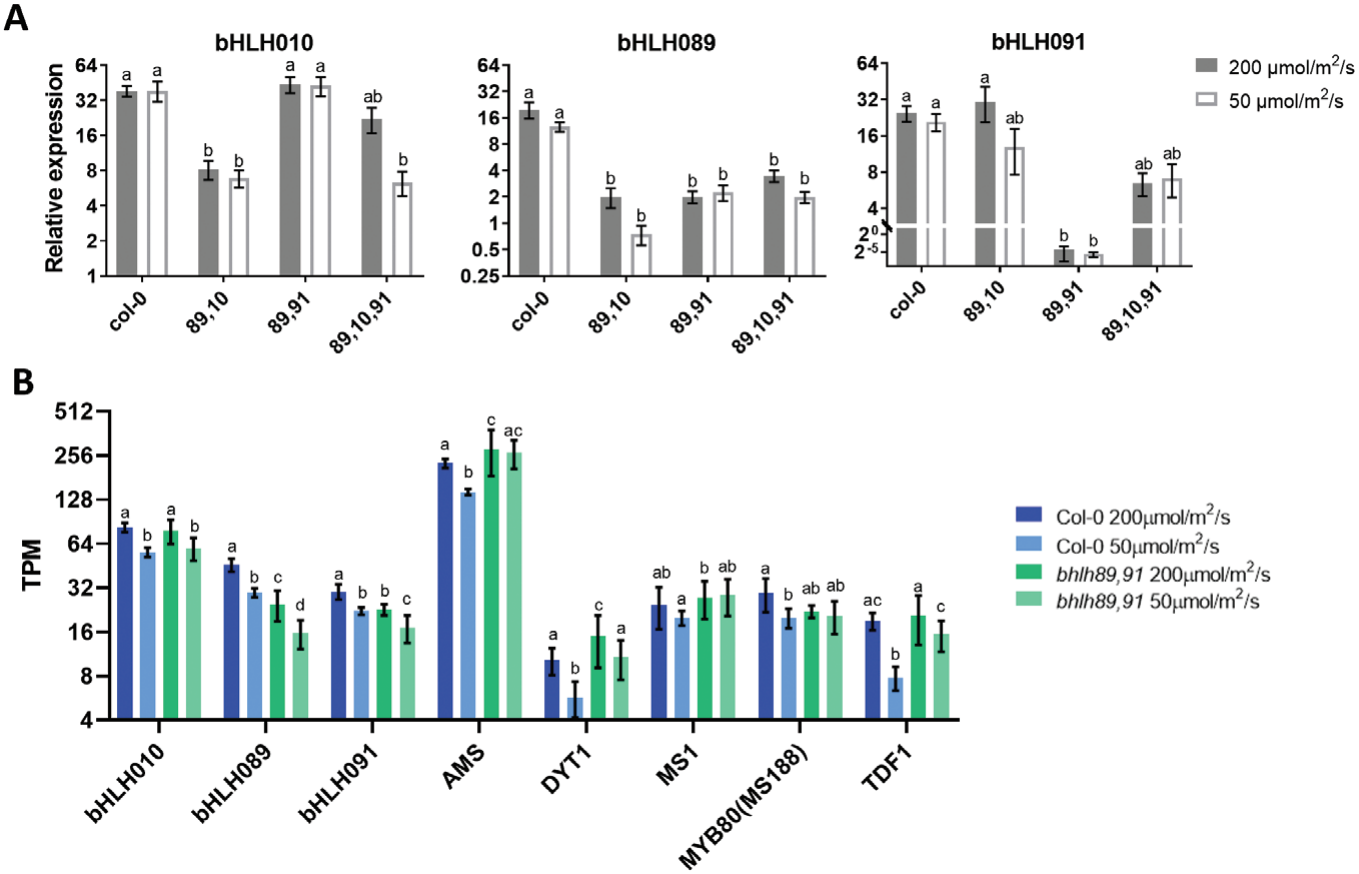
Gene expression changes under altered environments and genetic backgrounds. (A) Relative expression of bHLH genes in Col-0 and *bhlh* mutant lines under normal (200 µmol m^–2^ s^–1^) and low (50 µmol m^–2^ s^–1^) light conditions, as determined by qRT–PCR. Significant differences in expression (*P*<0.05) are highlighted with letters according to two-way ANOVA with a Tukey post-hoc test. (B) The effect of light on expression of key tapetum transcription factors in Col-0 and *bhlh89,91* according to RNA-seq. Gene expression is shown as mean transcripts per million (TPM). Letters represent significant differential expression within each gene, *q*<0.05 according to Benjamini–Hochberg correction of the false discovery rate. Error bars represent the SE of four biological replicates.

Within the *bhlh* double mutants, the T-DNA inserts are all located in the first exon of the *bHLH* genes (Supplementary Fig. S2) and were expected to cause loss-of-function mutations. Expression of *bHLH10*, *89*, and *91* transcripts within each of the *bhlh* double and triple mutants under both light and temperature conditions was assessed through qRT–PCR. Under normal growth conditions, *bHLH89* and *bHLH91* levels were knocked down to 12% and 0.3%, respectively, of WT levels in the new *bhlh89,91* mutant background, whilst in the *bhlh89,10* mutant *bHLH10* was reduced to 23% and *bHLH89* to 14% (Fig. 4A). Transcriptional and translational spatio-temporal expression patterns were unchanged by low light (Supplementary Figs S12, S13). qRT–PCR expression analysis further suggested that knocking out two of the three *bHLH* genes does not alter the expression of the remaining *bHLH* [ANOVA, *F*(1,14)=0.41, *P*>0.05, Fig. 4A].

Transcriptomic analysis was conducted to investigate the molecular mechanisms behind the environmentally sensitive genetic male sterility (EGMS) in the more sensitive *bhlh89,91* mutant. RNA was isolated from pre-mitosis I stage buds from Col-0 and *bhlh89,91* plants grown under normal and low light conditions, and sequenced using the BGISEQ-500 platform (BGI), mapped to Arabidopsis thaliana Reference Transcript Dataset 2 (AtRTD2; Zhang *et al.*, 2017), and analysed using the 3D-RNASeq app (Guo *et al.*, 2019). This RNA-seq dataset supported qRT–PCR results as bHLH10 transcripts were unaltered from Col-0 in the *bhlh89,91* mutant (*q*>0.05; Supplementary Table S2).

*bHLH89* and *bHLH91* transcription appeared to be slightly reduced under low light in Col-0 and *bhlh89,10* backgrounds according to the qRT–PCR (Fig. 4A); however, this was not statistically significant. Within the RNA-seq data, *bHLH10*, *89*, and *91* expression is slightly but significantly reduced under low light conditions in Col-0, with log_2_FCs of –0.55, –0.62, and –0.43 for bHLH10, 89, and 91 respectively (*q*≤0.01; Fig. 4B; Supplementary Table S2), Furthermore this reduction is largely maintained in the *bhlh89,91* mutant background (*q*≤0.05), with respective log_2_FCs of –0.47, –0.51, and –0.41. Likewise, the major transcription factor genes involved in tapetum development—*DYT1*, *TDF1*, *AMS*, and *MS188*—were all down-regulated under low light in Col-0 (Fig. 4B; Supplementary Table S2). However, while there was an observed down-regulation in low light in the *bhlh89,91* mutant for *DYT1*, *TDF1*, *bHLH89*, *91*, and *10*, both *AMS* and *MS188* are unaffected (*q*>0.05). *MS1* expression seems to be independent of light intensity and exhibited no change in response to light in either Col-0 or the *bhlh89,91* mutant background.

### The *bhlh89,91* mutant has reduced transcriptomic response to low light

**Fig. 5.**
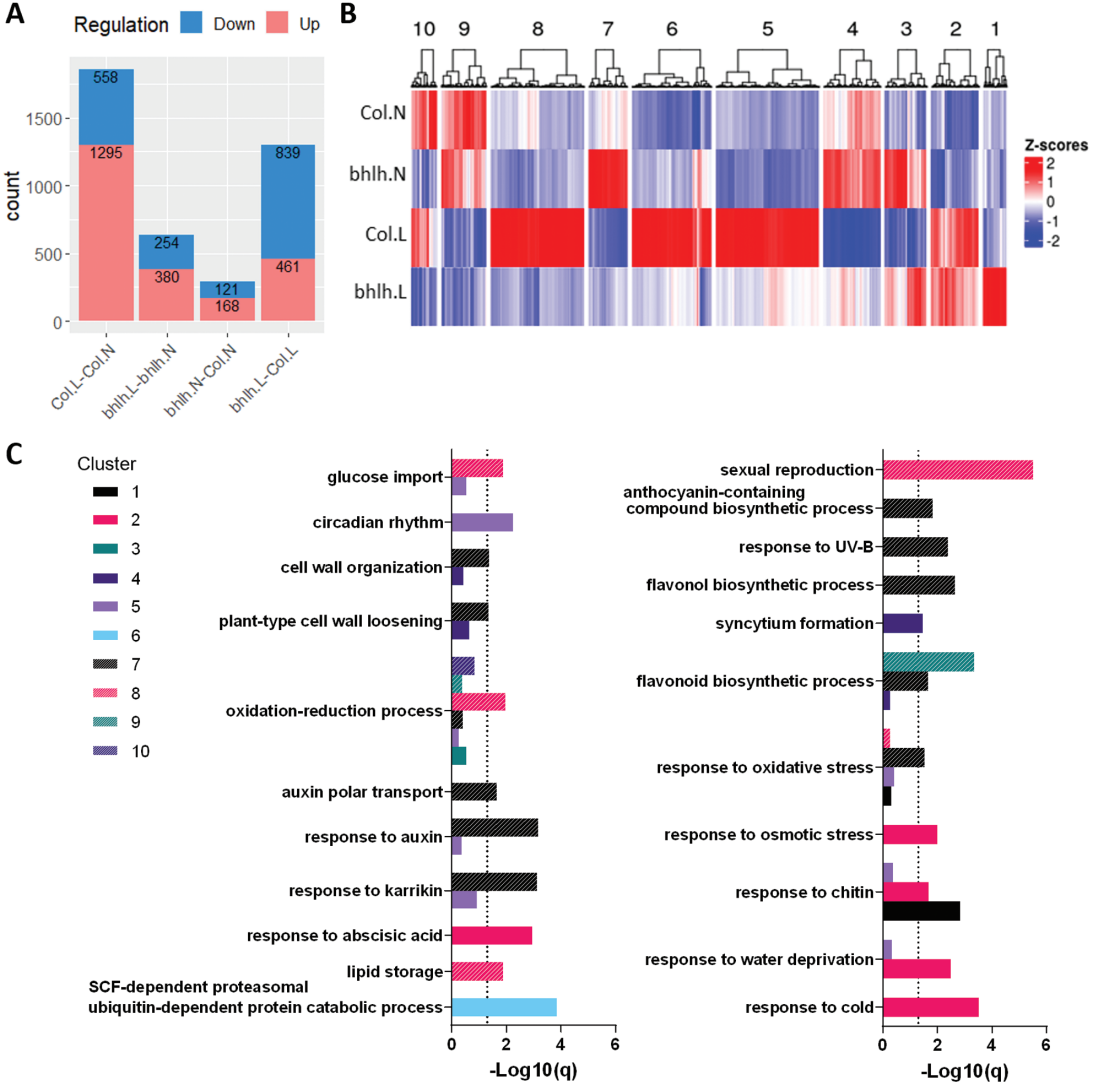
Differentially expressed genes (DEGs) in Col-0 and the *bhlh89,91* mutant under normal and low light conditions as determined by RNA-seq. (A) Number of up- and down-regulated DEGs for each contrast group. (B) Heatmap of DEGs, within each factor group, partitioned by hierarchical clustering with Ward’s algorithm and Euclidean distance. (C) Significant GO terms within each gene cluster are highlighted. Here *q* is the *P*-value adjustment by the Benjamini method, with a dotted line at *q*=0.05 representing the significance threshold.

RNA-seq analysis was carried out on total RNA isolated from pre-mitotic buds of Col-0 and *bhlh89,91* grown under normal and low light conditions. DEGs were identified with a log_2_FC≥1, and Benjamini–Hochberg FDR-adjusted *P*-value (*q*)≤0.01 (Fig. 5A; Supplementary Table S3). The *bhlh89,91* mutant showed a lower transcriptional response to light than the WT (Col-0), with 634 DEGs compared with 1853 DEGs in Col-0 (Fig. 5A). The *bhlh89,91* mutant transcriptome was vastly different from that of the WT under low light conditions, with 1300 genes differentially expressed from Col-0, compared with only 289 DEGs in normal light (Fig. 5A).

According to hierarchical clustering, DEGs cluster into 10 clades (Fig. 5B). Generally, clusters 2 and 9 contain genes that maintain a similar light response in both Col-0 and the *bhlh89,91* mutant, whereas genes in clusters 5, 6, and 8 show transcriptional activation in low light in Col-0 that is lost or reduced in *bhlh89,91*.

GO enrichment analysis (Fig. 5C) showed significant enrichment of GO terms such as sexual reproduction, lipid storage, glucose import, oxidation–reduction process, and SCF-dependent proteasomal ubiquitin-dependent catabolic process in clusters 5, 6, and 8, suggesting that these responses are impaired in the *bhlh89,91* mutant light response. However, clusters 2 and 9 show enrichment of responses to abscisic acid (ABA), cold, osmotic stress, and water deprivation and flavonoid biosynthetic process, suggesting that general stress responses are maintained in the *bhlh89,91* mutant.

KEGG pathway analysis of the DEGs further shows differential regulation (*q*≤0.05) of phenylpropanoid biosynthesis, biosynthesis of secondary metabolites, flavonoid biosynthesis, starch and sucrose metabolism, plant hormone signal transduction, and cutin, suberin, and wax biosynthesis in response to light (Supplementary Table S4). A closer look at these pathways highlights some major differences between Col-0 and *bhlh89,91* light responses (Supplementary Fig. S14). For example, within the phenylpropanoid biosynthetic pathway, spermidine biosynthesis shows differential gene expression in Col-0 in response to light but no equivalent changes are observed in the *bhlh89,91* mutant (Supplementary Fig. S14A). The plant hormone signal transduction pathway also shows up-regulation of key genes involved in auxin, cytokinin, and ABA signalling in Col-0 in low light that are altered, or absent, in the *bhlh89,91* mutant (Supplementary Fig. S14B, C).

To focus in on more direct effects of bHLH89 and 91 in light responses within the anther, we filtered annotated DEGs to 84 genes that are expressed in the anther according to laser capture expression data, and which undergo a log_2_FC≥2 in light-treated Col-0 but are unchanged in the *bhlh89,91* mutant (*q*>0.01; Supplementary Table S5). A number of these genes have been previously shown to impact male fertility (Table 1), including known direct targets of the tapetal bHLHs, such as EXTRACELLULAR LIPASES (EXLs) 4–6 (Xu *et al.*, 2014; Fu *et al.*, 2020).

**Table 1.** Differential expression of key genes with an established role in male fertility

Locus	Gene	Col.L– Col.N	bhlh.L– bhlh.N	bhlh.N–Col.N	bhlh.L–Col.L
AT4G27420	*ABCG9*	2.00			–1.43
AT1G75940	*BGLU20*	4.46			–3.79
AT3G26940	*CDG1*	2.89			–2.22
AT1G74540	*CYP98A8*	3.22			–5.17
AT1G74550	*CYP98A9*	4.60			–3.74
AT1G75910	*EXL4*	8.55			–6.77
AT1G75920	*EXL5*	4.46			–4.06
AT1G75930	*EXL6*	7.19			–4.25
AT5G28470	*FST1*	4.28			–4.47
AT5G59845	*GASA10*	2.02		1.96	
AT5G07530	*GRP17*	5.28			
AT3G47870	*LBD27*	3.13			–1.71
AT1G24400	*LHT2*	2.77			–2.01
AT1G18280	*LTPG3*	5.26			–2.98
AT4G08670	*LTPG4*	4.77			–2.38
AT2G32460	*MYB101*	2.81			–2.02
AT5G37810	*NIP4-1*	4.48			–4.27
AT5G59810	*SBT5.4*	2.67			–1.92
AT2G19070	*SHT*	2.20			–2.87
AT5G40260	*SWEET8*	2.20			–1.48
AT3G08790	*TOL8*	3.51			–2.44
AT1G67990	*TSM1*	2.67			–2.65
AT5G53550	*YSL3*	2.23			–2.35

Log_2_FC (*q*<0.01) values shown with blank cells represent missing/non-significant values.

## Discussion

### After the switch—light affects reproductive development beyond floral transition

Environmentally sensitive genetic male sterility (EGMS), with plants remaining male sterile under certain conditions and regaining fertility in a different environment, is a highly desirable trait for breeding crops with increased hybrid vigour (Kim and Zhang, 2018). Thermosensitive genetic male sterility (TGMS) and photoperiod genetic male sterility (PGMS) lines are well established in breeding practices; however, little is known about the effects of light levels on male reproductive development.

Light quality is known to affect the initiation of flowering, with a low red:far red (R:FR) light ratio activating floral transition via phytochrome-mediated pathways involving bHLH proteins: PHYTOCHROME INTERACTING FACTORS (PIFs) (Franklin, 2008; Wit *et al*., 2016). In light, phyA and phyB photoreceptors interact with PIFs, leading to their inactivation or degradation (Pham *et al*., 2018), whereas in shade or low R:FR light, this interaction ceases, enabling the PIFs to bind to targets and regulate pathways including skotomorphogenesis and floral transition.

Maintaining optimal light and temperature is vital across reproductive processes from sporogenesis to anthesis (Kinet, 1982; Pacini, 2000). Whilst the effect of temperature on anther development has been explored in great detail (Rieu *et al.*, 2017), very few studies have disentangled the effects of irradiance. Light, as a limiting factor in photosynthetic carbon fixation, is critical for all stages of plant development including reproduction (Ferguson *et al.*, 2021). The developing anther is metabolically active, with high energy demands and carbohydrate requirements (Clément *et al.*, 1996; Ji *et al.*, 2010; Zhang *et al.*, 2010). In suboptimal light conditions, photo-assimilation is reduced and resource allocation must be altered to maintain highly demanding reproductive processes. However, very little is known about the genetic regulation of this.

This study indicates that light influences reproductive development through bHLH89 and bHLH91. Both double mutants *bhlh89,91* and *bhlh89,10* show EGMS in response to light and heat. Under normal light conditions (200 µmol m^–2^ s^–1^), both mutants display an initial period of sterility, with fertility later restored to WT levels. However, under low light conditions (50 µmol m^–2^ s^–1^), there is a delay in this onset of fertility in *bhlh89,10*, while *bhlh89,91* has more pronounced defects with limited seed set (Fig. 1) and clumped non-viable pollen throughout development (Fig. 2; Supplementary Fig. S8). Minor effects of low light were also observed in the *bhlh89* and *bhlh91* single mutants. Further study at higher light levels suggests that this conditional fertility response is dynamic, with both double mutants showing a reduced period of sterility when grown under 300 µmol m^–2^ s^–1^ light (Supplementary Fig. S6).

The delay in the onset of fertility in the *bhlh89,10* mutant under normal light conditions is consistent with the observation of Zhu *et al.* (2015) of sterility in early *bhlh089 bhlh010* mutant flowers. However, this prolonged sterility also occurs in the *bhlh89,91* mutant. This phenotype may have not been observed by Zhu *et al.* as the T-DNA insert in their *bhlh091* mutant did not disrupt an exon (Supplementary Fig. S2). However, given the dynamic fertility of *bhlh* mutants in response to light (Fig. 1; Supplementary Fig. S6), it is also possible that the fertility defects may have been masked under different environmental conditions in the study of Zhu *et al.*

Since the *bhlh89,91* mutant shows more severe EGMS under low light conditions, and *bhlh89* and *bhlh91* single mutants also show some sterility (Fig. 1; Supplementary Fig. S4) this study suggests that, although a high level of functional redundancy has been reported among bHLH89, 91, and 10 (Cui *et al.*, 2016), and all appear to be involved in maintaining pollen development under increased heat (Fu *et al.*, 2020), bHLH10 plays little or no role in maintaining reproduction in response to suboptimal light. Therefore, we focused on *bhlh89,91* for RNA-seq analysis.

### bHLH89 and bHLH91 may integrate environmental signals into the tapetum development pathway upstream of DYT1

Our RNA-seq data show that the main transcription factors involved in anther development are affected by light. *DYT1*, *TDF1*, and *AMS* were down-regulated in response to low light in Col-0, but this response was reduced in the *bhlh89,91* mutant for *DYT1* and *TDF1*, and lost completely for *AMS*, suggesting that bHLH89/91 is required for their light regulation. *MS188* was down-regulated in low light only in Col-0 buds, and *MS1* exhibited no change in response to light in either background. This suggests that low light modulates the DYT1–TDF1–AMS–MS188 transcriptional cascade, with bHLH89/91 activity involved in maintaining the transcriptional response of *AMS* and *MS188* under adverse growth conditions. Notably, 18 of the direct targets of AMS previously identified (Xu *et al.*, 2014) are up-regulated in response to low light in Col-0 but not in the *bhlh89,91* mutant. This suggests that light may modulate bHLH89/91 co-activation of a large proportion of AMS direct targets, with a substantial knock-on effect on the downstream genes (Supplementary Table S3).

bHLH transcripts are slightly, but significantly, reduced in response to low light in both *bhlh89,91* and Col-0, with *bHLH89*, *91*, and *10* expressed at ~60–70% of normal levels. Given that *bHLH89*, *91*, and *10* have some degree of functional redundancy (Zhu *et al.*, 2015), it is possible that the reduced expression of the remaining functional bHLH within *bhlh* double mutants causes reduced fertility. However, the expression changes are relatively small, and there are no obvious differences in bHLH expression patterns in low light conditions (Fig. 4; Supplementary Fig. S12), thus this is unlikely to have such large effects on fertility. At the protein level, translational fusions suggest that bHLH proteins are even marginally increased under low light (Supplementary Fig. S13). As the tapetal bHLHs form dimers with distinct functions in regulating downstream genes (Cui *et al.*, 2016), post-translational mechanisms may also play a role here in regulating protein interactions and contribute to the different levels of conditional sterility within the two *bhlh* double mutants.

### The *bhlh89,91* mutant has a reduced transcriptional response to low light, with differential regulation of sexual reproduction processes

Under normal light, there are minimal differences between Col-0 and the *bhlh89,91* mutant transcriptomes, according to RNA-seq data. However, these differences are more than quadrupled under low light stress, primarily due to changes in transcription in low light in Col-0 that are absent in the *bhlh89,91* mutant (Fig. 5A). A number of biological processes appear to be impaired in the *bhlh89,91* mutant (Fig. 5C). Lipid storage genes, for example, are enriched in Col-0 but not *bhlh89,91* in response to low light; however, we could find no evidence of a changed lipid state in later stages of pollen development (Supplementary Fig. S11). Fitting with the phenotypic data, there is no GO term enrichment of genes related to sexual reproduction in the light response of the *bhlh89,91* mutant, suggesting that bHLH89 and bHLH91 may integrate environmental signals to control reproductive development in adverse conditions. Indeed, we identified a number of light-sensitive bHLH-dependent genes with a confirmed role in male reproductive development (Table 1).

Hormonal signalling and responses appear to be differentially regulated between the WT and *bhlh89,91* mutant. bHLH89 and 91 up-regulate key genes involved in auxin, cytokinin, and ABA signalling pathways under low light (Supplementary Fig. S14) and are involved in responses to auxin, jasmonic acid (JA), and gibberellic acid (GA) (Fig. 5C; Supplementary Fig. S15). Reduced signalling in a number of these phytohormone pathways could underlie the sterility observed under low light. Auxin synthesis, for example, plays a major role in stamen and pollen development (Cheng *et al.*, 2006; Cecchetti *et al.*, 2008), with auxin transport from the tapetum to the middle layer vital in maintaining fertility (Cecchetti *et al.*, 2017). Whilst we know that this is controlled by a number of genes, it is possible that the tapetal bHLHs relay environmental signals into these genetic pathways. GA has long been linked to male fertility; double and triple mutants of the redundant GA biosynthesis genes GA20OX1, 2, and 3 exhibit sterile phenotypes similar to *bhlh010*, *089*, and *091* mutants. The *ga20ox1ga20ox2* double mutant is semi-fertile with impaired seed set in early flowers and reduced silique elongation (Rieu *et al.*, 2008; Plackett et al., 2012). Whilst our RNA-seq data point towards some regulation of hormonal pathways by bHLH89 and 91, further studies are required to establish whether reduced fertility is caused by altered hormonal signalling.

### bHLH089 and bHLH091 may regulate pollen wall development in response to light

Among the direct AMS targets differentially regulated in response to light, EXL4 is strongly up-regulated in response to low light in the WT but not the *bhlh89,91* mutant. The EXL4 gene encodes a sporophytic pollen coat protein essential for pollen wall formation (Updegraff *et al.*, 2009) and has been shown to be directly regulated by both AMS (Xu *et al.*, 2014) and DYT1 in combination with bHLH89 (Fu *et al.*, 2020). Network analysis of light-responsive DEGs shows that EXL4 forms a central hub within the largest subnetwork of the interactome (Supplementary Fig. S16), pulling together key genes involved in pollen wall development. This may suggest that altered bHLH89/91 protein interactions with AMS and DYT1 under low light directly regulate pollen wall formation.

Recently Lai *et al.* (2022) demonstrated that *bHLH89* and *bHLH10* are required for normal formation of the pollen exine and intine, through positive regulation of the phospholipid and flavonol metabolic pathways. The data we present herein suggest that bHLH91, in addition to bHLH89 and bHLH10, might also play an important role in regulating pollen wall development, specifically in response to altered environmental conditions, with both *bhlh89,10* and *bhlh89,91* double mutants exhibiting defective pollen wall patterning under low light conditions (Fig. 2). GO enrichment and KEGG pathway analysis suggested that spermidine biosynthesis and metabolism change in Col-0 in response to light but not in the *bhlh89,91* mutant (Supplementary Figs S14A, S15). Polyamines, such as spermidine, are involved in abiotic and biotic stress adaptations as well as normal plant growth and reproductive development, with losses of biosynthetic enzymes resulting in decreased pollen viability (Chen *et al.*, 2014). Four of the bHLH-dependent and light-responsive DEGs highlighted in this study (Table 1) affect the levels of spermidine in the pollen coat. TAPETUM-SPECIFIC METHYLTRANSFERASE 1 (TSM1), the BAHD acyltransferase SPERMIDINE HYDROXYCINNAMOYL TRANSFERASE (SHT), and two partially redundant cytochrome P450 enzymes CYP98A8 and CYP98A9 (Fellenberg *et al.*, 2008; Grienenberger *et al.*, 2009; Matsuno *et al.*, 2009) all act together in a single spermidine biosynthetic pathway in pollen development (Fellenberg *et al.*, 2009). Grienenberger *et al.* (2009) hypothesized that the location of these spermidines in the outer layer of the pollen coat may protect the pollen grain against environmental stresses. Since we observed differential regulation of these four enzymes in response to low light stress in the *bhlh89,91* mutant, we might expect major changes in spermidine derivatives in the pollen coat compared with Col-0, leading to reduced protection of the pollen from environmental stresses. It may be that up-regulation of these genes by bHLH89 or 91 allows the pollen to retain structure despite adverse environmental conditions. Loss of these spermidines may be contributing to the collapsed pollen seen under low light in the *bhlh* double mutants (Fig. 2).

In addition to spermidines, it is likely that bHLH89 and 91 regulate pollen development more generally. Recent work by Truskina *et al.* (2022) highlighted the role of intercellular signalling pathways and subtilases in coordinating tapetum and pollen grain development. SBT5.4, which is differentially regulated by bHLH89/91 in response to light (Table 1), was found to be crucial in synchronizing organized secretion of pollen wall components into the anther locule (Truskina *et al.*, 2022). Ectopic expression of SBT5.4 leads to defective exine patterning, and clumped pollen similar to that observed under low light in the *bhlh89,91* mutant (Fig. 2F).

These *bhlh89,91* pollen abnormalities under low light are also reminiscent of the *abcg9,abcg31* mutant in which pollen grains shrivel and clump together upon exposure to air (Choi *et al.*, 2014). These two ATP binding cassette (ABC) transporters, involved in the deposition of steryl glycosides on the pollen coat, are down-regulated in the *bhlh010 bhlh089* mutant (Lai *et al.*, 2022) and, according to our own data, appear to be regulated by bHLH89/91 in response to light (Supplementary Table S3). Lai *et al.* demonstrated that ABCG9 is directly activated by bHLH89. Since both ABCG9 and ABCG31 are up-regulated in Col-0 responses to low light, but are unchanged in the *bhlh89,91* mutant, this further suggests that pollen coat deposition is directly regulated in response to environmental signals through bHLH89/91.

In conclusion, we have shown that bHLH89 and bHLH91 play an important role in regulating fertility in response to light, with the *bhlh89,91* and *bhlh89,10* mutants showing varying degrees of reduced fertility under low light characterized by defective pollen wall formation and non-viable pollen. This supplements recent work on the importance of bHLH89 and 10 in regulating pollen exine development (Lai *et al.*, 2022) and maintaining fertility under high temperature stress (Fu *et al.*, 2020), suggesting that bHLH89, 10, and 91 may play a broad role in mitigating environmental variation to ensure that pollen development and fertility are maintained. We found that low light induces major transcriptional changes in the early stages of flower development in Col-0 that are absent in the *bhlh89,91* mutant. This indicates that bHLH89 and bHLH91 are critical in floral response to low light stimulus, through regulation of genes related to sexual reproduction, lipid storage and metabolism, and hormonal responses. Further analysis of the control and function of these genes will help to elucidate the role of the bHLHs in male reproductive development in response to a changing environment and provide new avenues for understanding how environmental signals control pollen development, with potential to be adopted into EGMS breeding practices.

## Supplementary data

The following supplementary data are available at *JXB* online.

Fig. S1. qRT–PCR of the housekeeping gene PP2A3.

Fig. S2. Location of T-DNA inserts in *bhlh.*

Fig. S3. Correlation of seed number and silique length.

Fig. S4. Fertility analysis of *bhlh* single mutants.

Fig. S5. Spectra of light treatments.

Fig. S6. Effect of high light levels on silique length in *bhlh* double mutants.

Fig. S7. Comparison of bud sizes at each stage of microspore development in Col-0 and *bhlh* mutant inflorescences.

Fig. S8. Alexander viability staining of pollen under different light intensities.

Fig. S9. TEM sections of Col-0 and the *bhlh89,91* double mutant.

Fig. S10. Semi-thin sections of Col-0 and *bhlh89,91* double mutant anthers in low light conditions.

Fig. S11. Nile red staining of tricellular pollen from Col-0 and *bhlh* double mutants.

Fig. S12. X-Gluc staining of bHLH transcriptional reporter inflorescences and dissected anthers.

Fig. S13. Expression of translational bHLH florescent reporter lines under normal and low light.

Fig. S14. KEGG pathway changes in the *bhlh89,91* double mutant.

Fig. S15. GO enrichment of biological processes in Col-0 and the *bhlh89,91* in normal versus low light DEGs.

Fig. S16. Network analysis of light-responsive DEGs in Col-0.

Table S1. Primers for qRT–PCR expression analysis.

Table S2. Expression of key tapetum transcription factors within RNA-seq.

Table S3. Total differentially expressed genes (DEGs).

Table S4. KEGG pathway enrichment of DEGs.

Table S5. 84 annotated anther-expressed DEGs.

erad480_suppl_Supplementary_Tables_S1-S5

erad480_suppl_Supplementary_Figures_S1-S16

## Data Availability

Sequence data from this article can be found in the National Center for Biotechnology Information Gene Expression Omnibus under series number GSE248735. AMS (AT2G16910), bHLH10 (AT2G31220), bHLH89 (AT1G06170), bHLH91 (AT2G31210), DYT1 (AT4G21330), MS1 (AT5G22260), MS188/MYB80 (AT5G56110), TDF1 (AT3G28470).
