# Supplementary material for: Environmental regulation of male fertility is mediated through Arabidopsis transcription factors bHLH89, 91, and 10
Source: J Exp Bot. 2023 Dec 8;75(7):1934–47. doi: 10.1093/jxb/erad480 (PMC10967248; doi:10.1093/jxb/erad480)
Supplement: erad480_suppl_Supplementary_Figures_S1-S16 [file erad480_suppl_supplementary_figures_s1-s16.pdf]

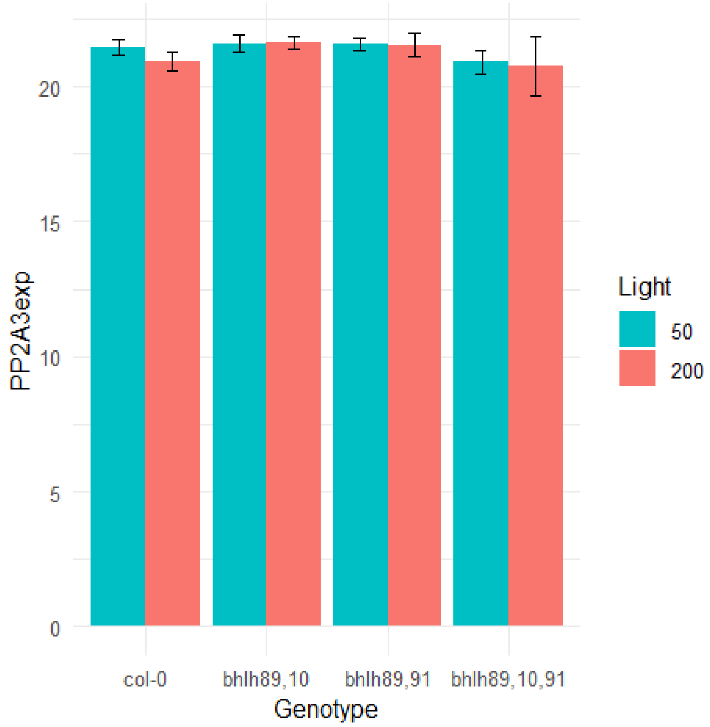

Figure S1. qRT-PCR showing expression of the housekeeping gene PP2A3 in the different lines and environmental conditions used in this study

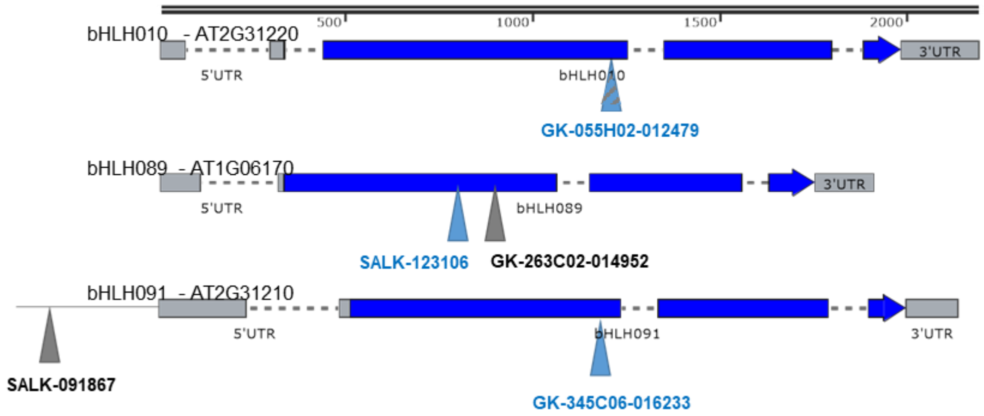

Figure S2. Location of T-DNA inserts in bHLH sequences, blue triangles represent location of inserts used in this study, all are located in exons (blue boxes). GK-055H02-012479 (*bhlh010*) was used in both (blue and grey triangle), while the grey triangles represent the location of inserts used in Zhu *et al.* (2015), and for the creation of the triple mutant *bhlh089,bhlh010,amiR-bHLH091*.

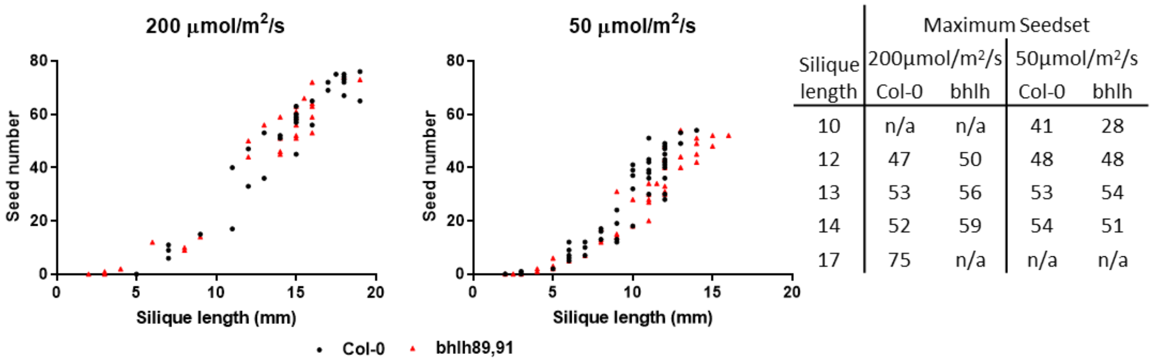

Silique length	Maximum Seedset 200µmol/m²/s Col-0	200µmol/m²/s bhlh	50µmol/m²/s Col-0	50µmol/m²/s bhlh
10	n/a	n/a	41	28
12	47	50	48	48
13	53	56	53	54
14	52	59	54	51
17	75	n/a	n/a	n/a

Figure S3. Correlation of seed number and silique length, within individual siliques of Col-0 and *bhlh89,91* in each light condition. Siliques longer than 10mm in low-light treated *bhlh89,91* represent occasional, and possibly outcrossed fertile siliques.

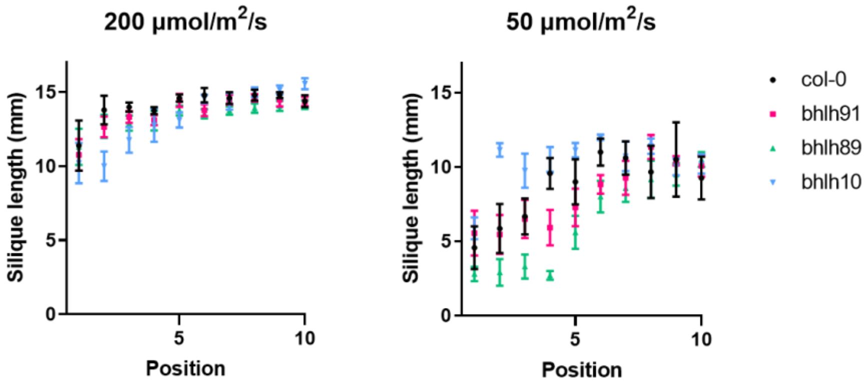

Figure S4. Fertility analysis of *bhlh* single mutants showing the effect of low light (50 µmol/m^2/s), measured by silique length across the initial 10 flower positions. Mean values and standard error of the mean plotted.

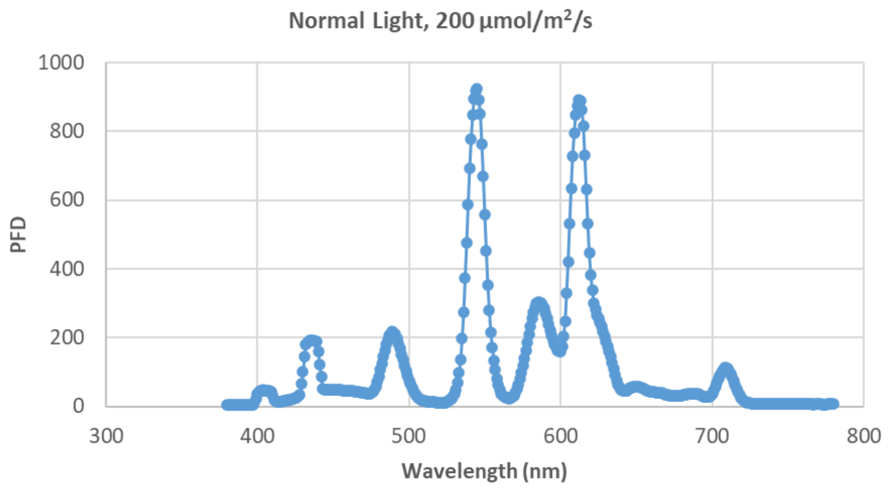

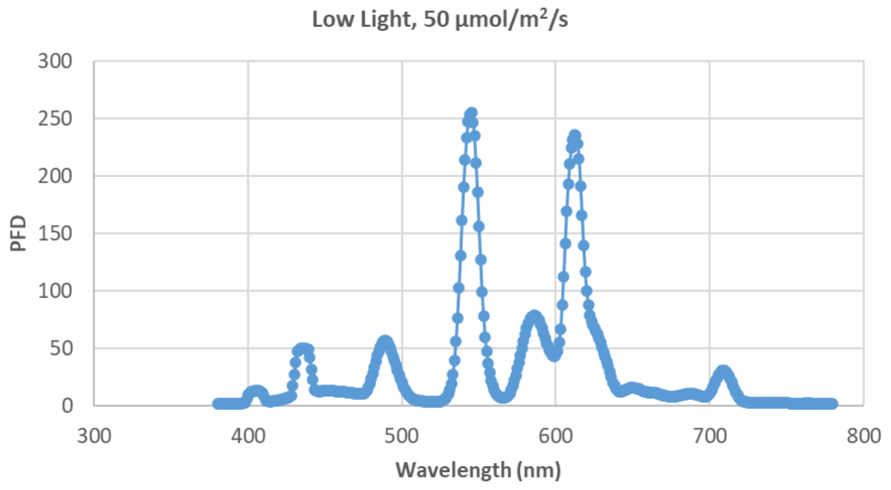

Figure S5. Spectra of normal and low light treatments with Photon Flux Density (PFD) plotted against wavelength, shows that whilst intensity is changed between light treatments, spectral quality is consistent. Recorded using Li-180 Spectrometer (Licor) at rosette level.

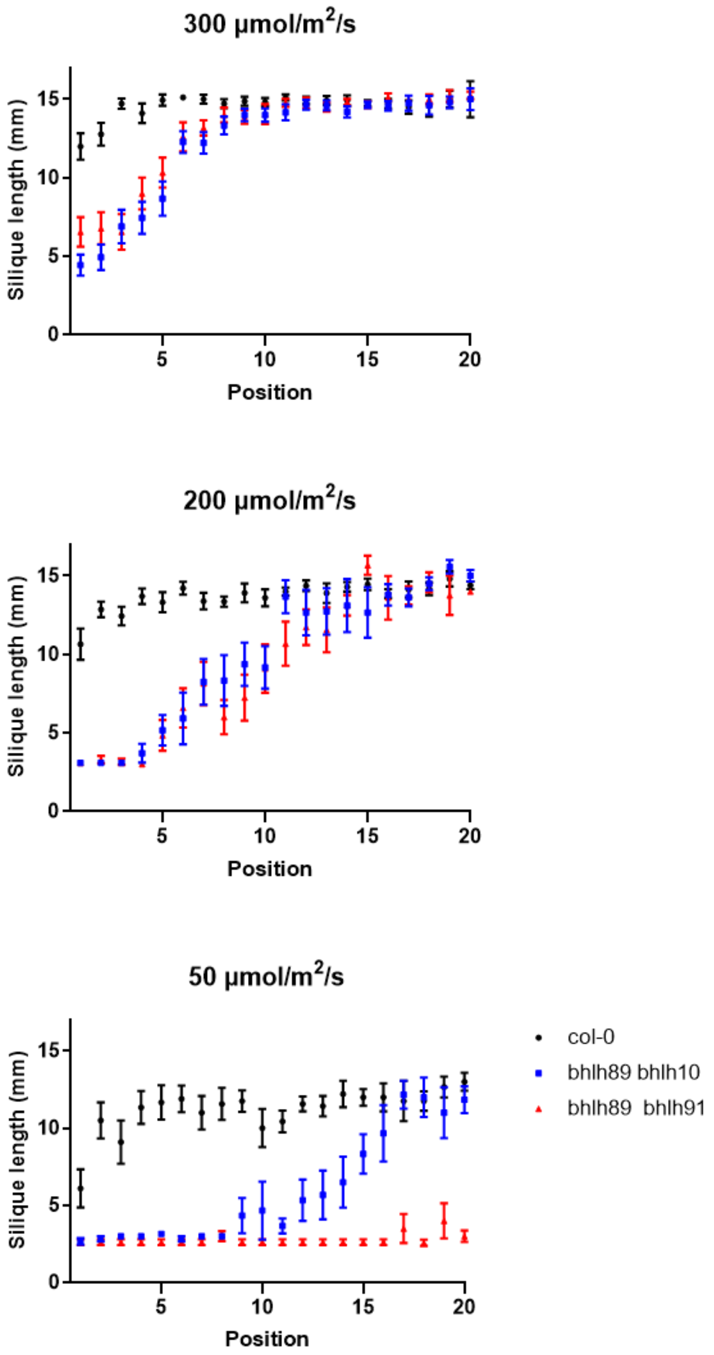

Figure S6. Characterisation of *bhlh* double mutants. The effect of high light (300 µmol/m^2/s), normal light (200 µmol/m^2/s) and low light (50 µmol/m^2/s), measured by silique length across the flowering stem, showing a reduction in initial sterility under high light and prolonged initial sterility in low light for the *bhlh* double mutants. Mean values and standard error of the mean plotted.

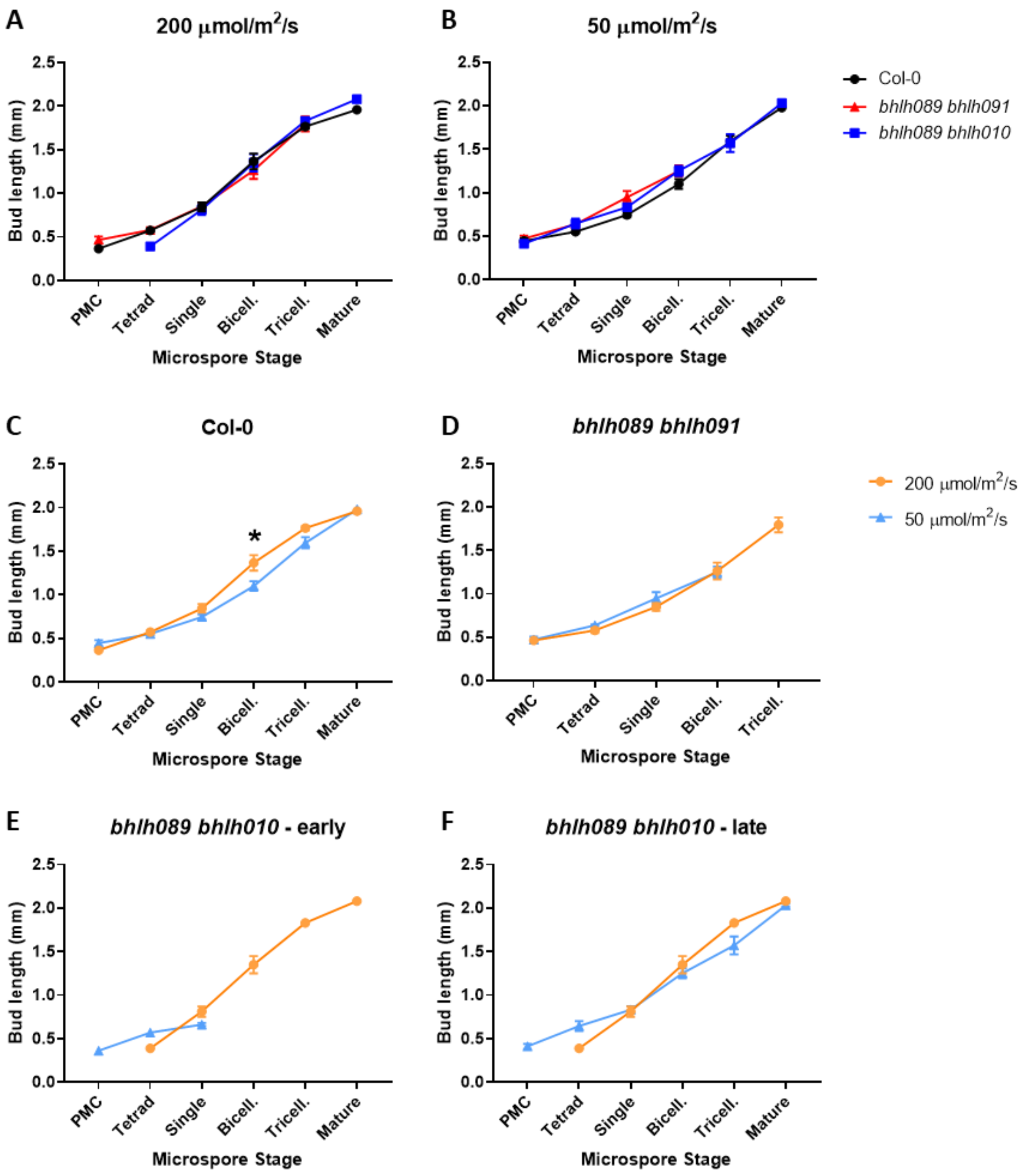

Figure S7. Comparison of bud sizes at each stage of microspore development in Col-0 and *bhlh* mutant inflorescences grown under (A) 200 and (B) 50 µmol/m^2/s light. The effect of light on bud size at different developmental stages is shown for (C) Col-0, (D) *bhlh089 bhlh091* and (E,F) *bhlh089 bhlh010*. A-D,F represent 'late' inflorescences, taken after 20+ flowers have already opened, whereas E is from 'early' inflorescences at the beginning of the flowering stem with less than 3 open flowers. Mean and SEM plotted. Asterisk indicates significant difference according to a student's T-test ($p<0.05$).

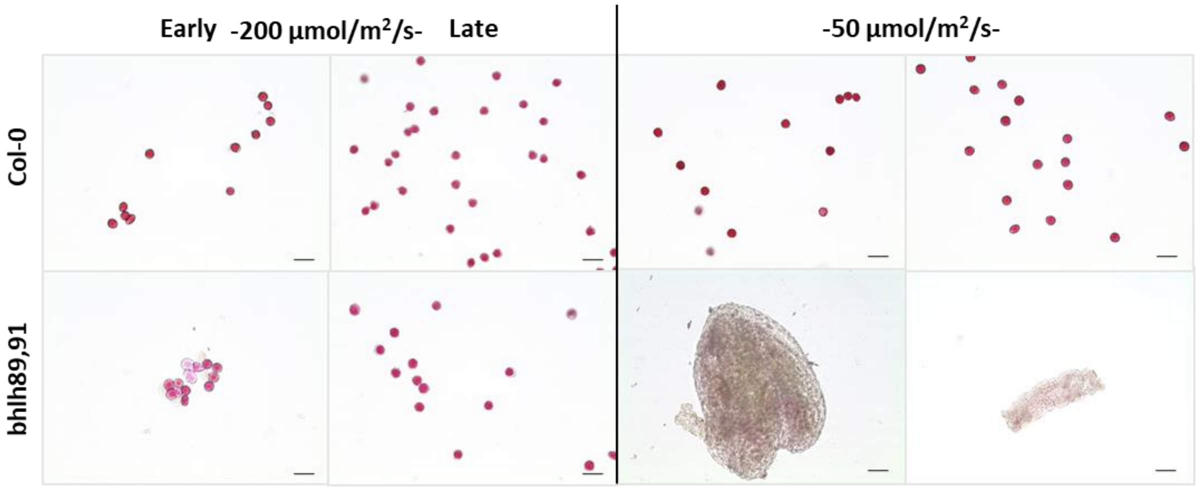

Figure S8. Alexander viability staining of pollen under different light intensities. Pink is viable, while pale pink/colourless is non-viable pollen. Scale bar 50µm.

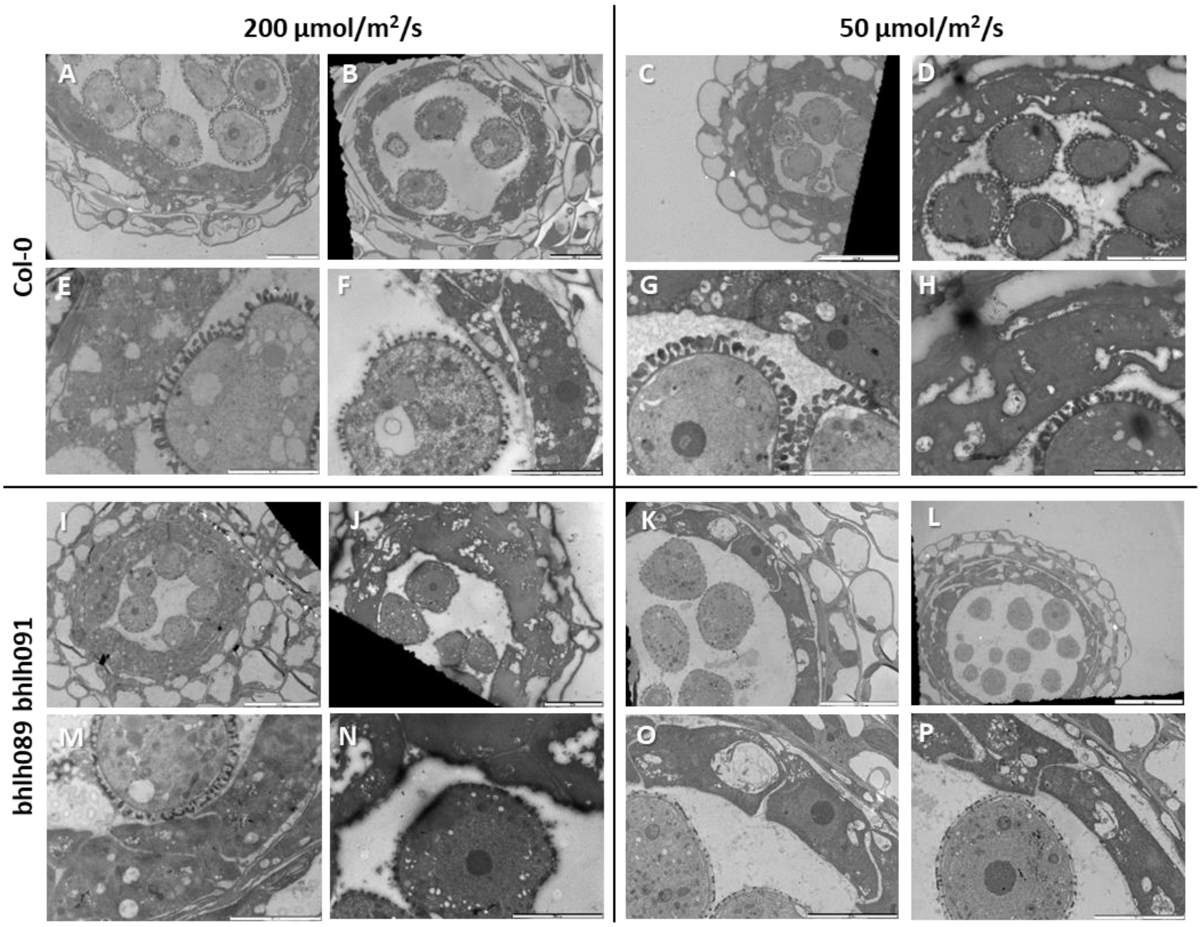

Fig. S9. TEM sections of WT (A-H) and *bhlh089 bhlh091* (I-P) single microspore stage anthers grown under normal and low light conditions. Scale bar: 1000µm for A,B,C,J,K; 2000µm for D,I,L,; 5000µm for E-H,M-P

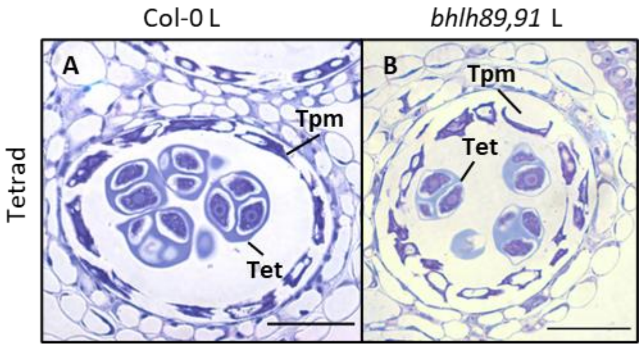

Figure S10. Semi-thin sections of Col-0 (A) and *bhlh89,91* double mutant (B) anthers, in low (L) light conditions at tetrad stage of microspore development. Tpm, tapetum; Tet, tetrads. Scale bar 50µm.

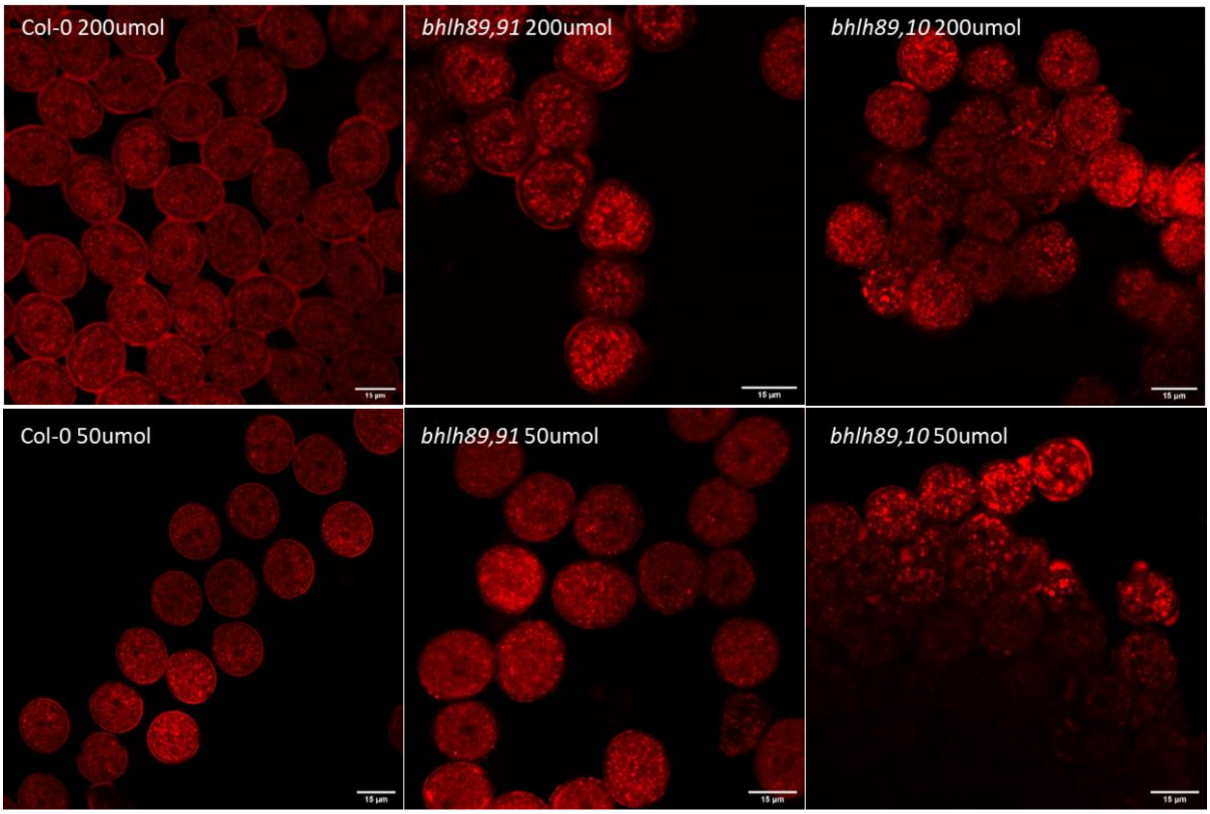

Figure S11. Nile red staining of tricellular pollen from Col-0 and *bHLH* double mutants grown under different light conditions, showing lipid body distribution. Nile red stains polar membrane lipids deep red, whilst neutral lipid in intracellular storage fluoresce yellow. Scale bar 15µm.

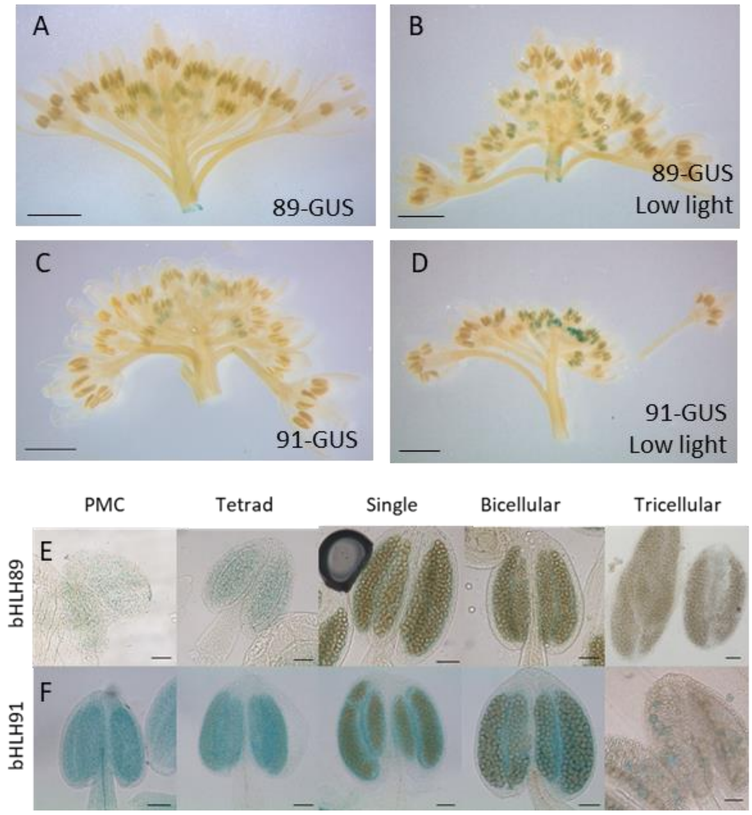

Figure S12. X-Gluc staining of bHLH transcriptional reporter inflorescences (A-D) and dissected anthers (E-F) showing spatial-temporal patterns of bHLH gene expression. pbHLH::GUS expression is prolonged from Pollen Mother Cell Stage to Bicellular Microspore stage in the tapetum with some GUS expression also seen in Tricellular Microspores of pbHLH91:GUS (F). pbHLH:GUS expression patterns persist under low light treatment (B,D). Scale Bars represent 1mm in A- D and 50µm in E-F.

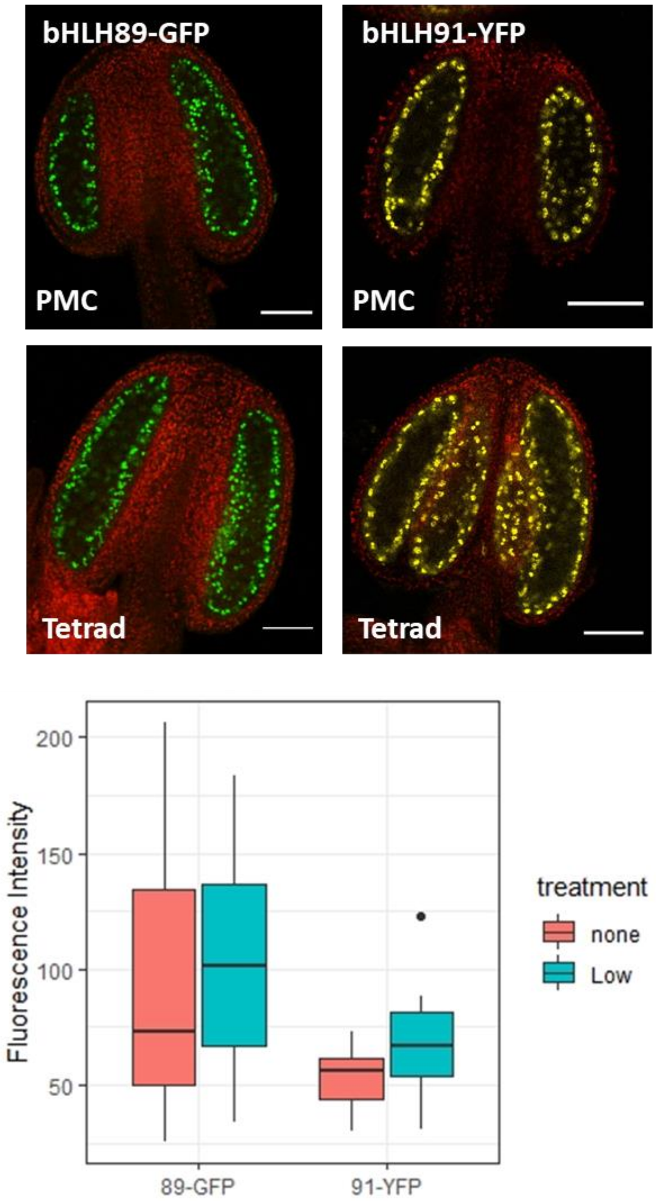

Figure S13. Expression of translational bHLH-fluorescent reporter proteins in WT backgrounds. bHLH89-GFP and bHLH91-YFP expression in the nuclei of tapetum tissue at Pollen Mother Cell (PMC) and Tetrad stages of anther development. Fluorescence Intensity plot showing bHLH- fluorophore response to low light. Scale bars represent 50µM

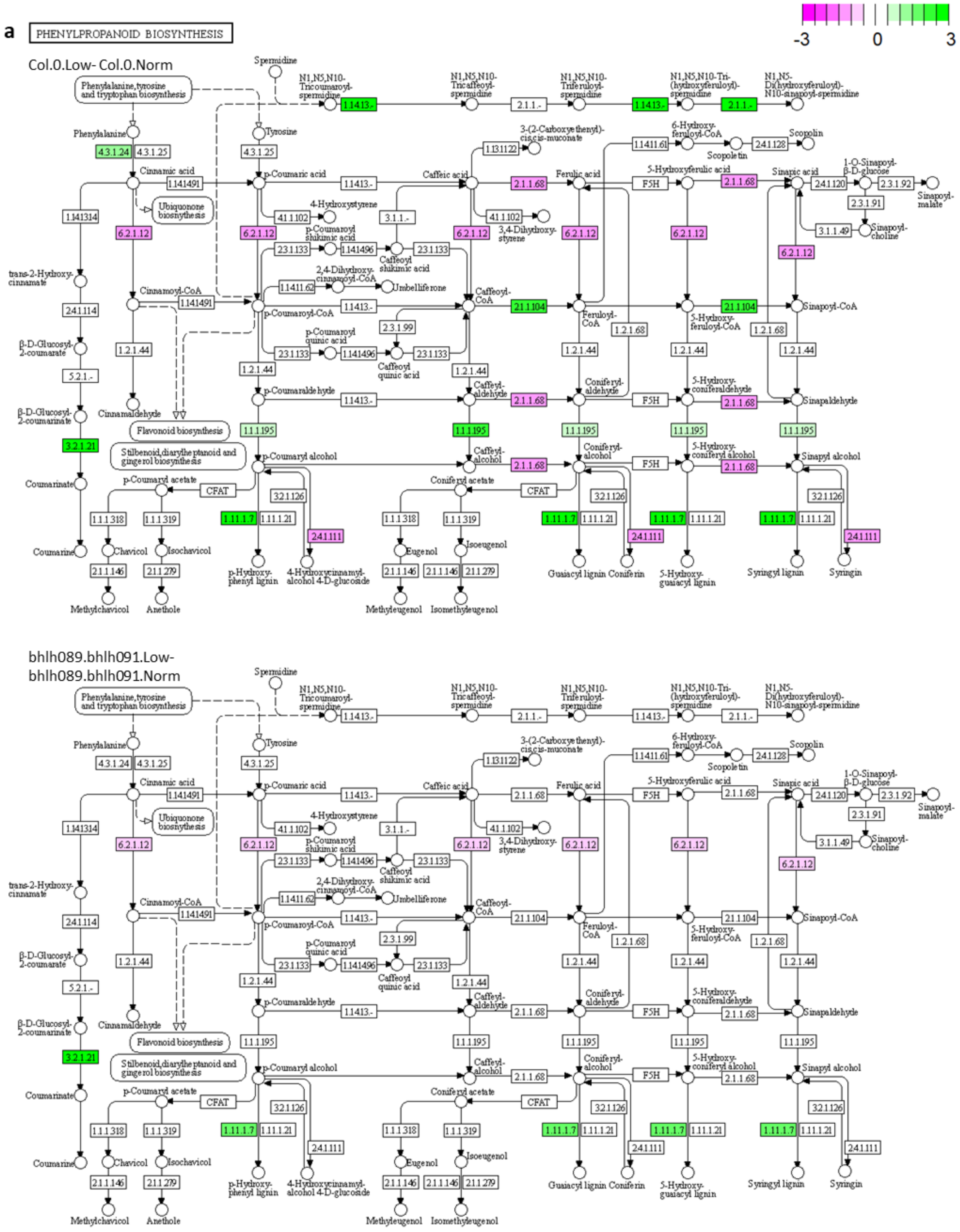

Figure S14A. KEGG pathway changes the *bhlh89,91* mutant; Phenylpropanoid biosynthesis fill colour showing log2FC of low-light upregulated (green) and downregulated (magenta) DEGs in Col-0 (top) and bhlh89,91 mutant (bottom). Cut-off for significant DEGs log2FC>1, q<0.01. Plot created using Pathview in R.

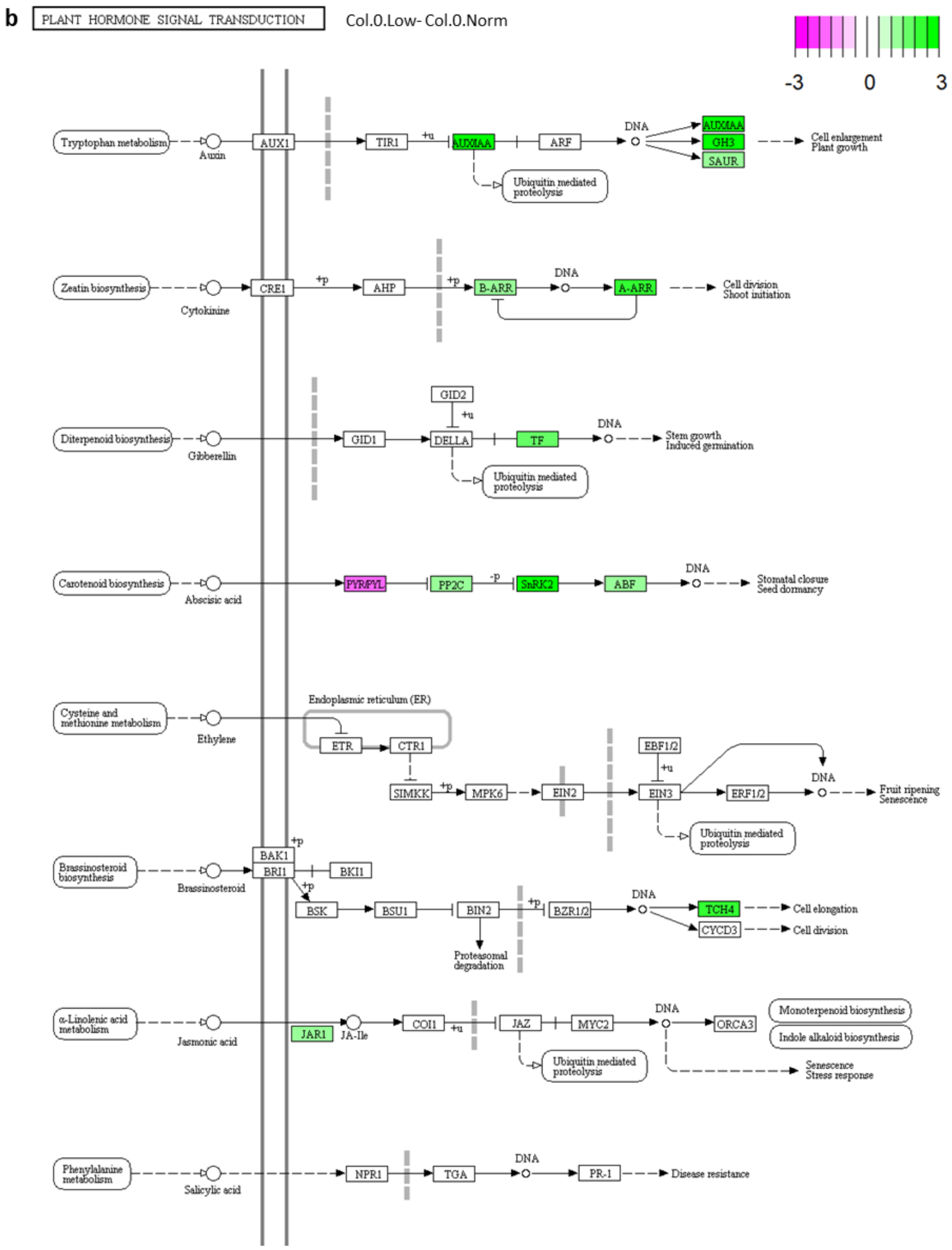
b
PLANT HORMONE SIGNAL TRANSDUCTION
Col.0.Low- Col.0.Norm
-3
0
3
Tryptophan metabolism
Auxin
AUX1
TIR1
+u
AUX/IAA
ARF
DNA
AUX/IAA
GH3
SAUR
Cell enlargement
Plant growth
Ubiquitin mediated proteolysis
Zeatin biosynthesis
Cytokinine
CRE1
+p
AHP
+p
B-ARR
DNA
A-ARR
Cell division
Shoot initiation
GID2
+u
Diterpenoid biosynthesis
Gibberellin
GID1
DELLA
TF
DNA
Stem growth
Induced germination
Ubiquitin mediated proteolysis
Carotenoid biosynthesis
Abscisic acid
PYR/PYL
PP2C
-p
SnRK2
ABF
DNA
Stomatal closure
Seed dormancy
Endoplasmic reticulum (ER)
Cysteine and methionine metabolism
Ethylene
ETR
CTR1
SIMKK
+p
MPK6
EIN2
EBF1/2
+u
EIN3
ERF1/2
DNA
Fruit ripening
Senescence
Ubiquitin mediated proteolysis
+p
BAK1
BRI1
BKI1
Brassinosteroid biosynthesis
Brassinosteroid
+p
BSK
BSU1
BIN2
+p
BZR1/2
DNA
TCH4
CYCD3
Cell elongation
Cell division
Proteasomal degradation
α-Linolenic acid metabolism
Jasmonic acid
JAR1
JA-Ile
COI1
+u
JAZ
MYC2
DNA
ORCA3
Monoterpenoid biosynthesis
Indole alkaloid biosynthesis
Ubiquitin mediated proteolysis
Senescence
Stress response
Phenylalanine metabolism
Salicylic acid
NPR1
TGA
DNA
PR-1
Disease resistance

c

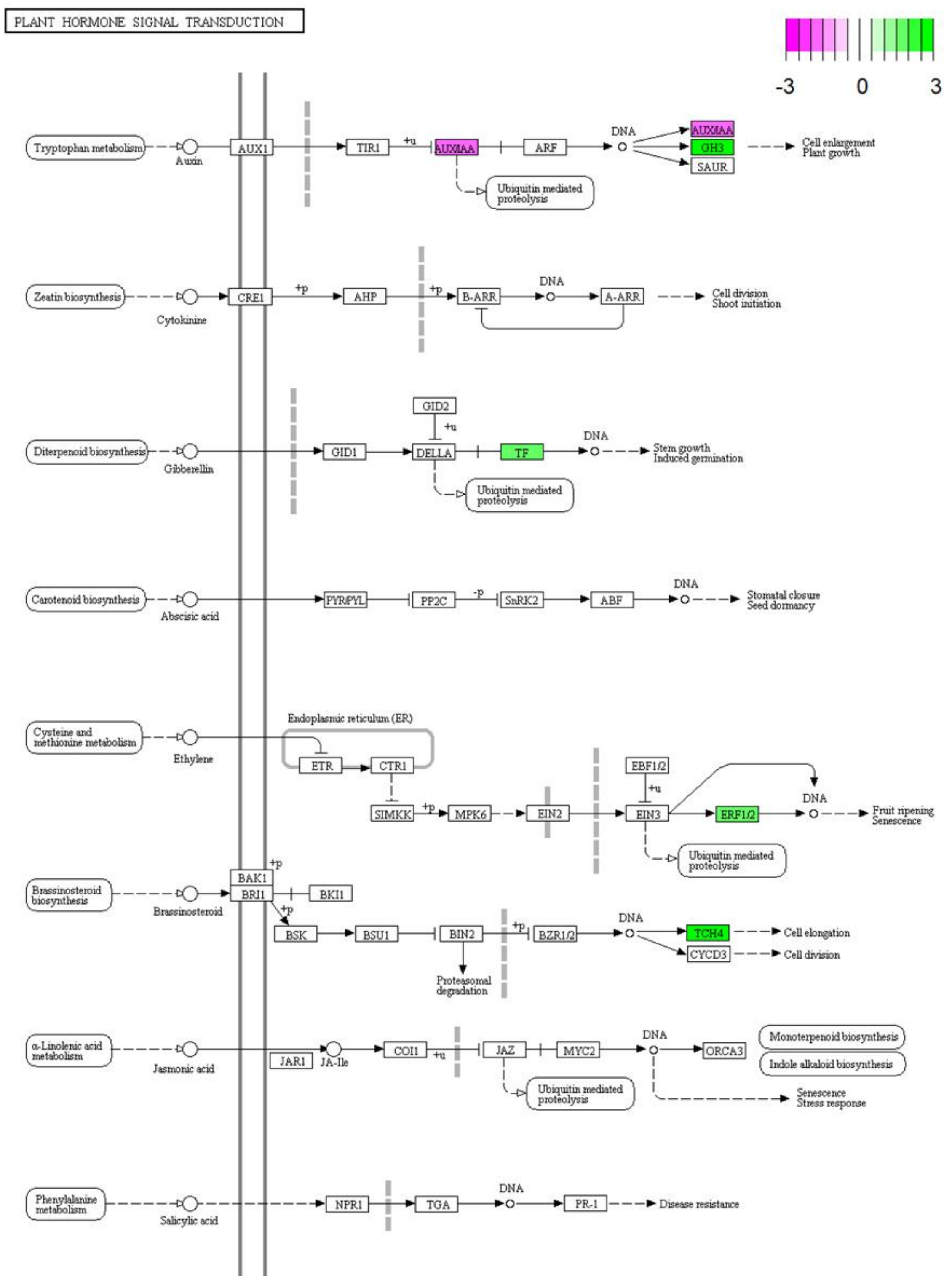

Figure S14B-C, continued from previous page. Plant Hormonal Signal Transduction KEGG pathway showing changes in DEGs involved in Auxin, Cytokinin, ABA and JA signalling pathways in low light in Col-0 that are absent or altered in the bhlh089 bhlh091 mutant. Fill colour intensity is indicative of low-light upregulated (green) and downregulated (magenta) DEGs in Col-0 (top) and bhlh89,91 mutant (bottom). Cut-off for significant DEGs log2FC>1, q<0.01. Plot created using Pathview in R.

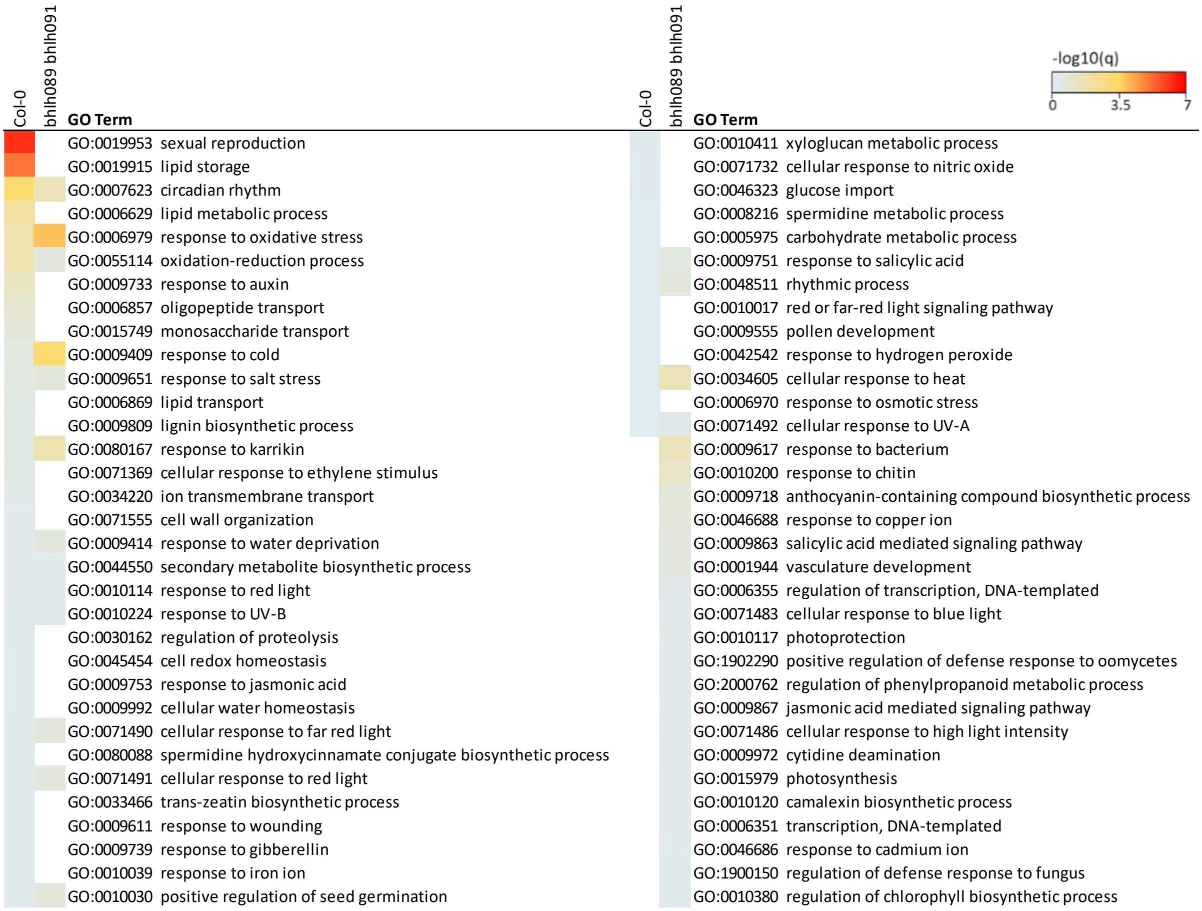

Figure S15. GO enrichment of biological processes in Col-0 and *bhlh089 bhlh091* normal vs low light DEGs (log_2FC>2). Colour scale represents -log10(q) where q is the p-value adjusted by the Benjamini method.

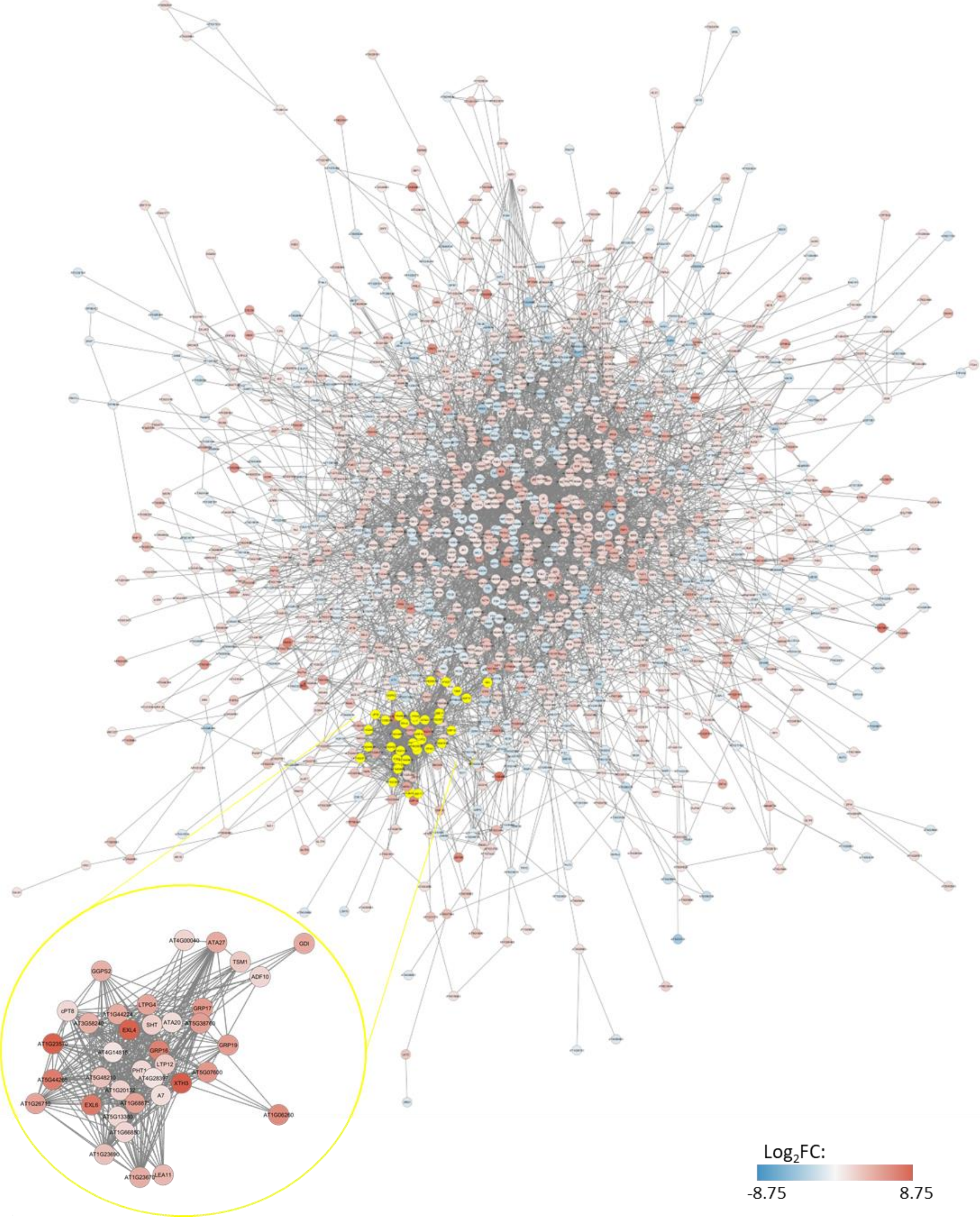

Fig S16. Network analysis of light responsive DEGs in Col-0. The largest subnetwork shows a condensed interactome centralised around EXL4, a direct target of bHLHs. EXL4 neighbour nodes are highlighted and inset.
